# Gallium complex K6 inhibits colorectal cancer by increasing ROS levels to induce DNA damage and enhance phosphatase and tensin homolog activity

**DOI:** 10.1002/mco2.665

**Published:** 2024-07-24

**Authors:** Wei Li, Chuanyu Yang, Zhuo Cheng, Yuanyuan Wu, Sihan Zhou, Xiaowei Qi, Yi Zhang, Jinhui Hu, Mingjin Xie, Ceshi Chen

**Affiliations:** ^1^ Yunnan Key Laboratory of Animal Models and Human Disease Mechanisms Kunming Institute of Zoology Chinese Academy of Sciences Kunming China; ^2^ Kunming College of Life Sciences University of Chinese Academy Sciences Kunming China; ^3^ School of Chemical Science and Technology Yunnan University Kunming China; ^4^ Department of Breast and Thyroid Surgery Southwest Hospital The First Affiliated Hospital of the Army Military Medical University Chongqing China; ^5^ The First Hospital of Hunan University of Chinese Medicine Changsha China; ^6^ Academy of Biomedical Engineering Kunming Medical University Kunming China; ^7^ The Third Affiliated Hospital Kunming Medical University Kunming China

**Keywords:** colorectal cancer, deoxyribonucleic acid (DNA) damage, gallium complex k6, phosphatase and tensin homolog (PTEN), reactive oxygen species (ROS)

## Abstract

Colorectal cancer (CRC) is one of the most common malignancies worldwide. In the clinical realm, platinum‐based drugs hold an important role in the chemotherapy of CRC. Nonetheless, a multitude of patients, due to tumor protein 53 (*TP53*) gene mutations, experience the emergence of drug resistance. This phenomenon gravely impairs the effectiveness of therapy and long‐term prognosis. Gallium, a metallic element akin to iron, has been reported that has the potential to be used to develop new metal anticancer drugs. In this study, we screened and established the gallium complex K6 as a potent antitumor agent in both in vitro and in vivo. K6 exhibited superior efficacy in impeding the growth, proliferation, and viability of CRC cells carrying *TP53* mutations compared to oxaliplatin. Mechanistically, K6 escalated reactive oxygen species levels and led deoxyribonucleic acid (DNA) damage. Furthermore, K6 effectively suppressed the phosphoinositide 3‐kinase (PI3K)/protein kinase B (PKB)/glycogen synthase kinase 3 beta (GSK3β) pathway, leading to the degradation of its downstream effectors myelocytomatosis (c‐Myc) and Krueppel‐like factor 5 (KLF5). Conversely, K6 diminished the protein expression of WW domain‐containing protein 1 (WWP1) while activating phosphatase and tensin homolog (PTEN) through c‐Myc degradation. This dual action further demonstrated the potential of K6 as a promising therapeutic compound for *TP53*‐mutated CRC.

## Graphical abstract

K6 inhibits CRC independent of *TP53* by inducing DNA damage and apoptosis, and by downregulating c‐Myc to activate PTEN.

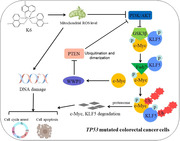

## 1 INTRODUCTION

Colorectal cancer (CRC) is a widespread malignant tumor in the digestive tract, both the incidence and mortality rates rank third globally, posing a substantial threat to human life and health.^1^ Surgical resection and chemotherapy are widely used in the treatment of CRC.^2^ Platinum drugs, such as oxaliplatin (L‐OHP), are the mainstay of CRC chemotherapy. Clinically, 5‐fluorouracil, leucovorin and L‐OHP (mFOLFOX6)/capecitabine and L‐OHP (CapeOX) with bevacizumab as first‐line therapy to treat metastatic CRC can significantly prolong the survival time of advanced CRC patients without surgical indications.^3^ Mechanistically, the tumor‐suppressing effect of L‐OHP is partially attributed to its capacity to induce cell cycle arrest and apoptosis, which arise from deoxyribonucleic acid (DNA) damage and activate the p53 pathway.^4^, ^5^ Nevertheless, approximately 40%−60% of CRC patients carry tumor protein 53 (*TP53*) gene mutations, which greatly affects the therapeutic efficacy of L‐OHP.^6^ In addition, CRC patients have serious adverse reactions due to the lack of tumor selectivity and low bioavailability of L‐OHP. These factors limit the application of L‐OHP in CRC treatment. Therefore, there is great significance to explore new reagents with anticancer activity that can target *TP53*‐mutated tumors and minimize damage to normal tissue cells to improve the survival of CRC patients.

Gallium (Ga) is a metallic element similar to iron, which is located in group IIIA of the fourth cycle on the periodic table, bearing the atomic number 31. As early as 1970s, ^67^Ga(III) was used in medical applications for tumor localization diagnosis. Significantly, gallium nitrate marked the inaugural approval by the Food and Drug Administration for a Ga(III) compound in addressing cancer‐related hypercalcemia.^7^ Interestingly, Hart and Adamson revealed that gallium nitrate has strong tumor‐suppressing activity both in vitro and in vivo, encompassing breast cancer, non‐Hodgkin's lymphoma and lung cancer.^8^ At present, an increasing number of new gallium complexes have been synthesized, and their antitumor activities have been reported, including Tris(8‐quinolinolato) gallium (KP46) and gallium maltolate, characterized by enhanced anticancer efficacy and superior oral bioavailability in comparison to gallium nitrate.^9^, ^10^ Balancing iron levels is vital for cell growth, survival, and biochemical processes to function optimally.^11^ As the chemical properties of Ga(III) and iron(III) are similar to each other. Ga(III) could bind with transferrin and transferrin receptor to interfere with the intake and utilization of iron by tumor cells and inhibit cell proliferation.^12^ In addition, Ga(III) can also induce mitochondrial oxidative stress, leading to apoptosis.^13^ Therefore, Ga(III) has the potential to be used to develop new metal anticancer drugs.

Myelocytomatosis (c‐Myc) protein is a prominent oncogenic transcription factor.^14^, ^15^ Under normal conditions, both the transcriptional and translational levels of the c‐Myc gene are tightly controlled. In contrast, p53, which encoded by the *TP53* gene, serves as a prominent tumor‐suppressing transcription factor that restrained *c‐Myc* transcription.^16^, ^17^ *TP53* mutation results in elevated expression of c‐Myc. Moreover, the c‐Myc protein undergoes ubiquitination and subsequent degradation via the proteasome.^18^ The E3 ubiquitin ligase F‐box and WD repeat domain containing 7 (Fbw7) recognizes glycogen synthase kinase 3 beta (GSK3β)‐mediated phosphorylated c‐Myc and ubiquitinated c‐Myc for degradation.^19^, ^20^ The phosphoinositide 3‐kinase/protein kinase B (PI3K/AKT) signaling pathway is recognized for its ability to suppress GSK3β activity. Activated AKT directly phosphorylates GSK3β at S9, leading to its inactivation.^21^ However, in most tumors, the PI3K/AKT signaling pathway is frequently activated, which result in the abnormally high expression of the c‐Myc.

In addition to *TP53*, phosphatase and tensin homolog (PTEN) stands as another formidable tumor suppressor gene, encoding a protein possessing both protein and lipid phosphatase activities.^22^ PTEN protein is mostly distributed in the cytoplasm and mainly plays a phosphatase activity, which can transform phosphatidylinositol 3,4,5‐triphosphate (PIP3) into phosphatidylinositol 4,5‐bisphosphate (PIP2) to antagonize the activity of the PI3K/AKT pathway.^23^ Consequently, loss of cytoplasmic PTEN leads to a large amount of abnormal accumulation of PIP3, activating the downstream AKT signaling pathway, induces malignant cell proliferation and accelerates the occurrence and development of tumors.^24^ In general, the nuclear localization of PTEN is believed to interact with radiation‐sensitive mutant 51 protein (RAD51), regulate DNA homologous recombination repair, and maintain DNA stability to inhibit tumor occurrence, indicating that PTEN in the nucleus also exhibits tumor‐suppressive functions.^25^ However, Xie et al. reported that in breast cancer, neddylation of PTEN could induce its nuclear location and dephosphorylate the fatty acid synthase (FASN) protein, suppress the tripartite motif‐containing protein 21 (TRIM21)‐mediated ubiquitination and degradation of FASN, promote de novo fatty acid synthesis, and ultimately facilitate cell proliferation and metastasis.^26^

Ubiquitination is an important regulation for the stability of PTEN protein and phospholipase activity. Nedd4‐1 was the first documented E3 ubiquitin ligase to polyubiquitinate and degrade PTEN.^27^ Following this discovery, several other E3 ubiquitin ligases such as TRIM27, X‐linked inhibitor of apoptosis protein (XIAP), WW domain‐containing protein 2 (WWP2), and carboxyl terminus of Hsc70‐interacting protein (CHIP) have also been identified to polyubiquitinate and degrade PTEN.^28^, ^29^, ^30^, ^31^ Recently, Lee et al. demonstrated that *WWP1*, as a c‐Myc target gene, could polyubiquitinate PTEN and inhibit the membrane localization and dimerization of PTEN, which is essential for its antitumorigenic activity.^32^

Zhou et al. synthesized a series of (8‐hydroxyquinoline) Ga(III) complexes and found that they have antitumor activity in A549 and HCT116 cells.^33^, ^34^ In this study, we screened several gallium complexes and found that K6, which also contains ligand 8‐hydroxyquinoline, has the strongest anticancer activity against CRC in vitro and in vivo. Importantly, K6 could induce DNA damage and activate the mitochondrial‐dependent apoptosis pathway. Furthermore, K6 inhibited the PI3K/AKT/GSK3β pathway and facilitated c‐Myc, Krueppel‐like factor 5 (KLF5) degradation and CRC cells proliferation. On the other hand, K6 also decreased WWP1 protein level and activates PTEN by c‐Myc degradation. In summary, these results suggest that K6 is a promising potential therapeutic compound for CRC patients, particularly those harboring *TP53* mutations.

## 2 RESULTS

### 2.1 K6 inhibits CRC cell growth

**FIGURE 1:**
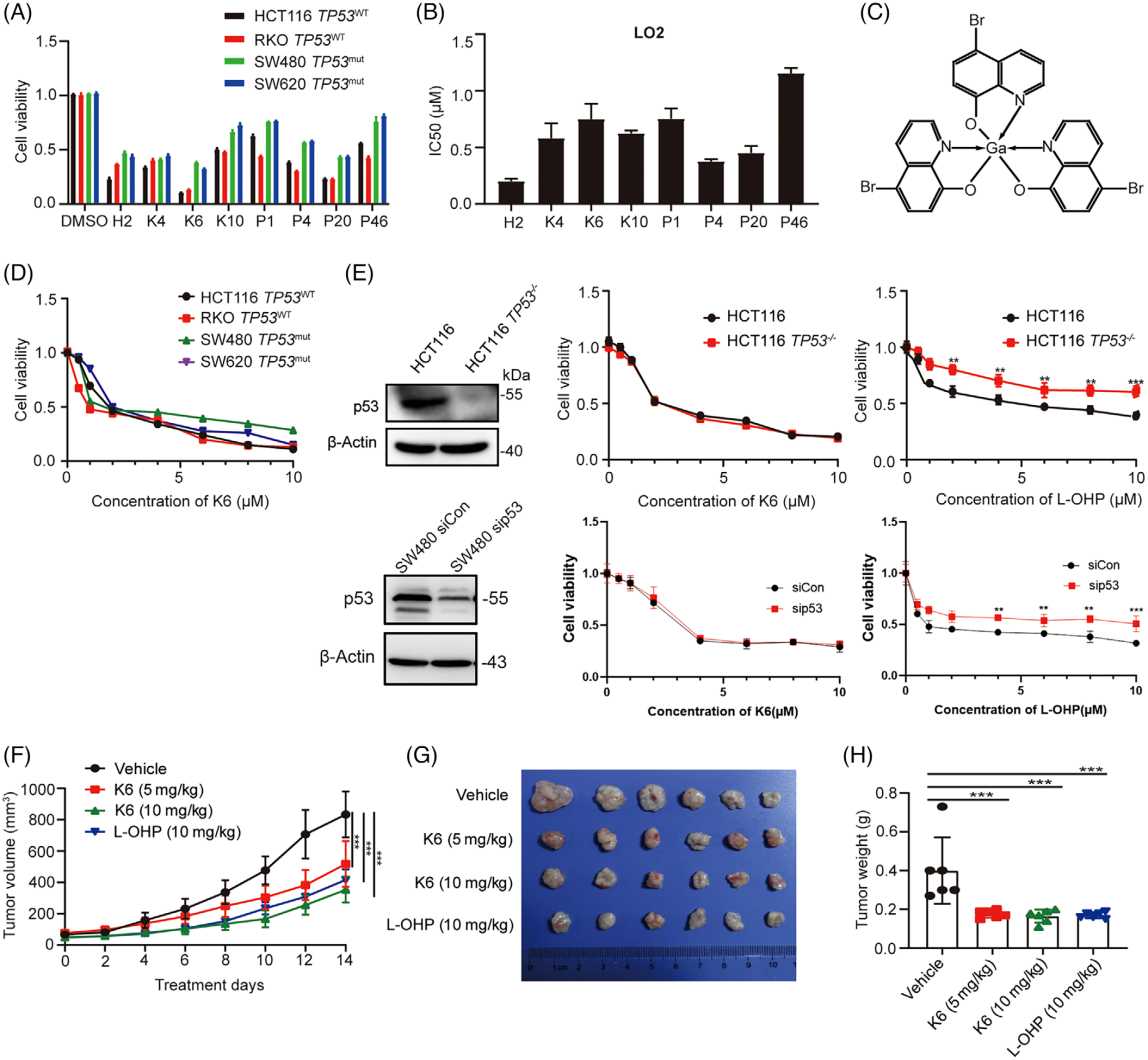
K6 inhibited the growth of colorectal cancer (CRC) cells. (A) The gallium complex K6 inhibited the activity of CRC cells the most. The percentage of cell viability after treatment with H2, K4, K6, K10, P1, P4, P20, or P46 at concentration of 10 µM for 48 h was assessed using the sulforhodamine B (SRB). (B) K6 had low toxicity in normal hepatocytes. The percentage of LO2 cell viability after treatment H2, K4, K6, K10, P1, P4, P20, and P46 at different concentrations for 48 h was evaluated via the SRB. (C) The chemical structure of K6. (D) K6 dose‐dependently reduced the cell viability of CRC cells. The percentage of cell viability following treatment with various concentrations of K6 for 48 h was determined using the SRB. (E) K6 did not depend on *TP53* to kill HCT116 CRC cells. The percentage of cell viability after treatment with K6 or oxaliplatin (L‐OHP) at concentrations of 0, 0.5, 1, 2, 4, 6, 8, or 10 µM for 48 h was assessed using SRB in HCT116 TP53^−/−^ or p53 transient knockdown SW480 cells. (F–H) K6 inhibited the subcutaneous growth of SW480 cells in nude mice. SW480 cells were subcutaneously injected into nude mice (3 × 10^6^/point, *n* = 6). When the tumor volume reached to 50−75 mm^3^, mice were administered K6 or L‐OHP by intraperitoneal injection. The tumor volume and weight were measured throughout the 2‐week treatment period.

To develop novel potential gallium complexes for the treatment of malignant tumors, we synthesized eight gallium complexes and compared their cytotoxicity in CRC cell lines with different *TP53* statuses (HCT116 *TP53* ^WT^, RKO *TP53* ^WT^, SW480 *TP53* ^mut^, SW620 *TP53* ^mut^). All cells were treated with each gallium complex (10 µM) for 48 h, and the cell viability was detected by sulforhodamine B (SRB) assay. As shown in Figure 1A, compared with other gallium complexes, K6 (Figure 1C) had the strongest ability to decrease the cell viability of CRC cells, and the toxicity of K6 in LO2 cells, a transformed hepatocyte that is often used to test toxicity of agents, was low (Figure 1B). Therefore, we further tested the toxicity of K6 at different concentrations in HCT116, RKO, SW480, and SW620 cells. We found that K6 decreased the cell viability of these cells in a dose‐dependent manner (Figure 1D). We noticed that the IC_50_ values were similar in four CRC cell lines, although SW480 and SW620 harbored *TP53* mutations.^35^ Furthermore, we found that K6 equally decreased the cell viability of HCT116 and HCT116 *TP53* ^−/−^ cells, although HCT116 *TP53* ^−/−^ cells were resistant to L‐OHP, which is consistent with transient knockdown of TP53 in SW480 cells (Figure 1E). Taken together, among the eight gallium complexes, K6 was the most promising gallium complex for antitumor in CRC cells, and its antitumor effect did not depend on *TP53* status.

Next, we evaluated the therapeutic potential of K6 to inhibit CRC in vivo. As demonstrated in Figure 1F–H, K6 significantly suppressed SW480 xenograft growth and reduced tumor weight compared to the vehicle control. It is worth noting that, compared with L‐OHP, which significantly reduced the body weight of mice, K6 had no substantial impact on the body weight of the animals (Figure S1A). Additionally, K6 also did not affect the levels of aspartate transaminase/alanine aminotransferase (AST/ALT) and creatinine (Cr), while L‐OHP significantly increased the level of Cr in serum (Figure S1B–D), suggesting that K6 has lower hepatorenal toxicity. We then conducted immunohistochemical staining for Ki‐67 in tumor tissue from SW480 tumor‐bearing mice and performed statistical analysis. The results showed that K6 decreased the levels of Ki‐67, c‐Myc, and PTEN in vivo (Figure S1E). To thoroughly validate these findings, we assessed the antitumor efficacy of K6 using the MC‐38 xenograft animal model. Our investigations revealed that K6 effectively suppressed tumor growth and notably extended the survival duration of MC38 tumor‐bearing mice (Figure S2A–H). These results suggested that K6 has anti‐CRC activity in vivo without obvious toxicity or side effects.

### 2.2 K6 inhibits cell proliferation and induces apoptosis of SW480 and SW620 cells

**FIGURE 2:**
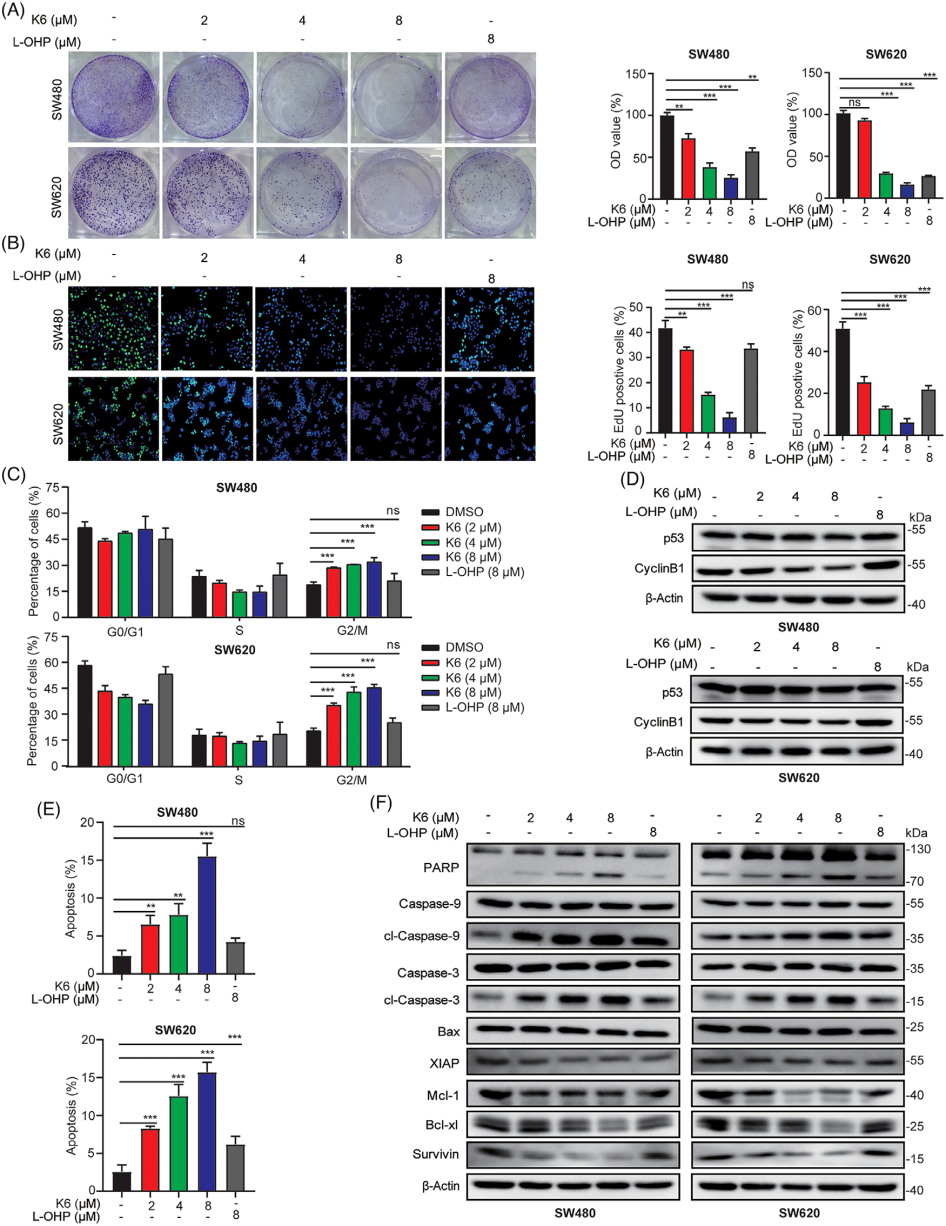
K6 inhibited the proliferation and induced apoptosis in SW480 and SW620 cell lines. (A) K6 inhibited the colony formation of SW480 and SW620 cells. Representative pictures and absorbance of SW480 and SW620 cells following treatment with K6 (2, 4, and 8 µM) or oxaliplatin (L‐OHP) (8 µM), respectively. (B) K6 inhibited deoxyribonucleic acid (DNA) synthesis in SW480 and SW620 cells. The DNA synthesis ability of SW480 and SW620 cells after treatment with K6 (2, 4, and 8 µM) or L‐OHP (8 µM) for 24 h was detected using a 5‐ethynyl‐2‐deoxyuridine (EdU) kit. (C) K6 induced G2/M phase cell cycle arrest in SW480 and SW620 cells. Cell cycle distribution in SW480 and SW620 cells following treatment with K6 (2, 4, and 8 µM) or L‐OHP (8 µM) for 24 h was analyzed via flow cytometry. (D) K6 inhibited the expression of cell cycle‐related proteins in SW480 and SW620 cells. Protein levels of CyclinB1 and p53 in SW480 and SW620 cells following treatment with K6 (2, 4, and 8 µM) or L‐OHP (8 µM) for 24 h were examined via Western blotting (WB). (E) K6 induced apoptosis of SW480 and SW620 cells. The cell apoptosis of SW480 and SW620 cells after treatment with K6 (2, 4, and 8 µM) or L‐OHP (8 µM) for 24 h was assessed using flow cytometry. (F) K6 promoted the expression of apoptotic proteins and inhibited the expression of proliferative proteins in SW480 and SW620 cells. The protein levels of poly ADP‐ribose polymerase (PARP), cl‐Caspase‐9/3, XIAP, Mcl‐1, Bcl‐xl, and Survivin in SW480 and SW620 cells after treatment with K6 (2, 4, and 8 µM) or L‐OHP (8 µM) for 24 h were determined using WB.

To evaluate the anti‐CRC activity of K6, we tested the effect of K6 on the cell proliferation of CRC cells. As shown in Figure 2A, K6 showed a remarkable ability to reduce the colony formation of the SW480 and SW620 cells. Then, we measured DNA synthesis using the 5‐ethynyl‐2‐deoxyuridine (EdU) incorporation assay. K6 inhibited the synthesis of DNA in a dose‐dependent manner (Figure 2B). Notably, the antitumor and DNA synthesis inhibition effect of K6 was significantly better than that of L‐OHP in both cell lines (Figure 2A,B). Subsequently, we subjected SW480 and SW620 cells to K6 or L‐OHP treatment for 24 h and performed cell cycle analysis via flow cytometry. As expected, K6 induced a significant dose‐dependent rise in the percentage of cells in the G2/M phase in both SW480 and SW620 cell lines (Figures 2C and S3A). Consistently, K6 decreased the protein expression levels of CyclinB1, which delayed the entrance of mitotic phase. Notably, the levels of p53 remained unaffected by K6 (Figure 2D). Next, we tested the effect of K6 on CRC cell apoptosis. SW480 and SW620 cells were stained with Annexin V and PI after 24 h of treatment with different concentrations of K6 or L‐OHP. By flow cytometry, K6 significantly increased the proportion of Annexin V‐ and PI‐positive apoptotic cells in a dose‐dependent manner, while the effect of L‐OHP on apoptosis was relatively weak (Figures 2E and S3B). Moreover, K6 induced the cleavage of Caspase‐9, the initial caspase in the mitochondrial apoptosis pathway, Caspase‐3 and PARP. Consistently, K6 dramatically reduced the protein expression levels of the antiapoptotic proteins XIAP, Mcl‐1, Bcl‐xl, and Survivin (Figure 2F). These data indicated that K6 significantly inhibited cell proliferation and promoted apoptosis by inducing mitochondrial apoptosis pathway activation in SW480 and SW620 cell lines.

### 2.3 K6 induces DNA damage by upregulating reactive oxygen species levels in SW480 and SW620 cell lines

**FIGURE 3:**
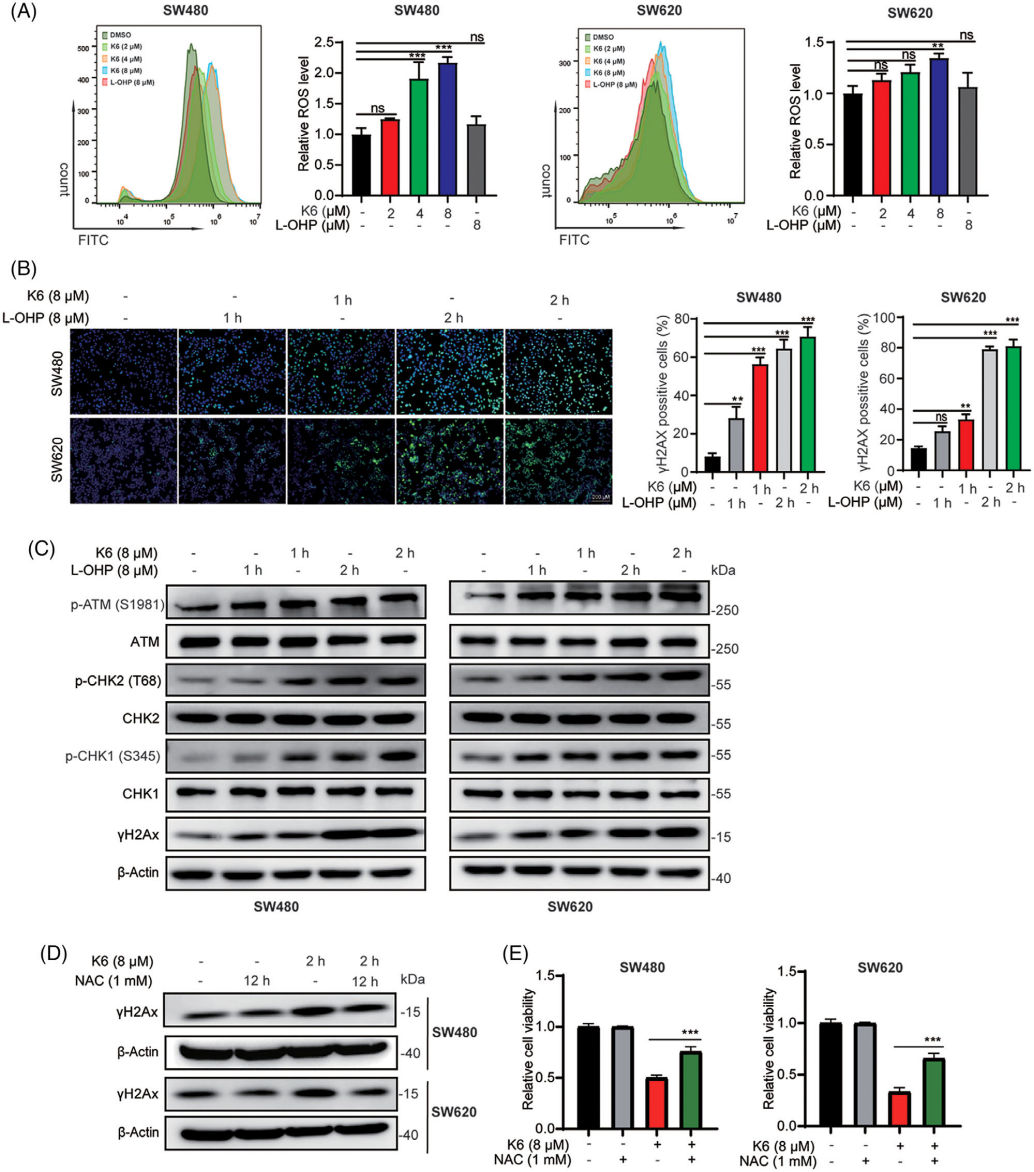
K6 upregulated reactive oxygen species (ROS) levels to induce deoxyribonucleic acid (DNA) damage in SW480 and SW620 cell lines. (A) K6 upregulated ROS levels in SW480 and SW620 cells. The ROS levels of SW480 and SW620 cells after treatment with K6 (2, 4, and 8 µM) or oxaliplatin (L‐OHP) (8 µM) for 6 h were assessed by flow cytometry. (B) K6 induced the rapid accumulation of γH2Ax in SW480 and SW620 cells. The γH2Ax accumulation levels in SW480 and SW620 cells after treatment with K6 (8 µM) or L‐OHP (8 µM) for 1 and 2 h were assessed by immunofluorescence. (C) K6 upregulated the phosphorylation of DNA damage‐related proteins in SW480 and SW620 cells. The phosphorylation levels of ATM, CHK1, CHK2, and H2Ax proteins in SW480 and SW620 cells after treatment with K6 (8 µM) or L‐OHP (8 µM) for 1 and 2 h were detected by Western blotting (WB). (D) N‐acetyl‐L‐cysteine (NAC) antagonized K6‐induced upregulation of γH2Ax protein levels. Following pretreatment of SW480 and SW620 cells with NAC (1 mM) for 12 h, cells were treated with K6 (8 µM) for 2 h, and γH2Ax protein levels were assessed by WB. (E) NAC inhibited the ability of K6 to downregulate the activity of SW480 and SW620 cells. SW480 and SW620 cells were treated with NAC (1 mM) or K6 (8 µM) alone or a combination of both for 48 h, and the cell viability was measured using sulforhodamine B (SRB).

Ga(III) has been reported to promote the generation of reactive oxygen species (ROS). Since K6 activates the mitochondrial apoptosis pathway, we speculated that K6 also upregulated ROS levels in CRC cells. As shown in Figure 3A, K6 increased ROS levels more strongly than L‐OHP in SW480 and SW620 cells. Excess intracellular ROS can induce DNA damage. Therefore, we evaluated DNA damage by measuring the levels of γ‐H2AX, a DNA damage marker. Remarkably, K6 treatment resulted in a rapid increase in γ‐H2AX (Figure 3B). Furthermore, K6 consistently increased the levels of p‐ATM, p‐CHK2, p‐CHK1, and γ‐H2AX in SW480 and SW620 cells in a time‐dependent manner (Figure 3C). To determine whether K6 induced DNA damage and apoptosis by upregulating the ROS level, we pretreated SW480 and SW620 cells with the antioxidant N‐acetyl‐L‐cysteine (NAC) for 12 h and then treated them with K6 for 2 h. As shown in Figure 3D, NAC significantly blocked the increase in γH2AX protein and rescued the loss of cell viability induced by K6 (Figure 3E). In culmination, these results provide that K6 induces DNA damage and cell death by increasing ROS levels in SW480 and SW620 cells.

### 2.4 K6 functions partially through inactivate the PI3K/AKT/GSK3β/c‐Myc pathway

**FIGURE 4:**
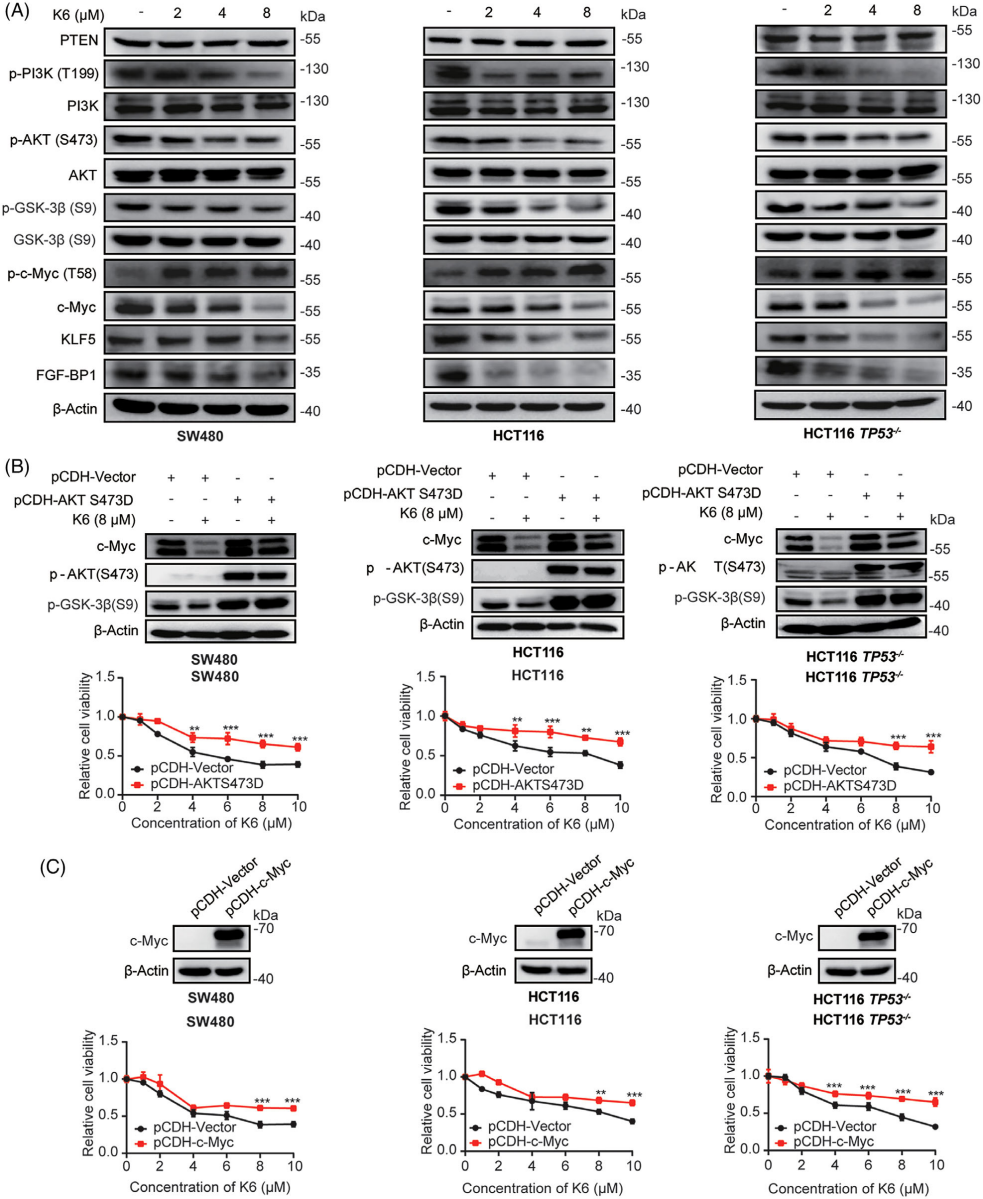
K6 functions partially through inactivate the phosphoinositide 3‐kinase/protein kinase B/glycogen synthase kinase 3 beta/myelocytomatosis (PI3K/AKT/GSK3β/c‐Myc) pathway. (A) K6 suppressed PI3K/AKT activity and the expression level of downstream proteins in colorectal cancer (CRC) cells. The activity of PI3K/AKT and the level of downstream proteins were examined by Western blotting (WB) in SW480, HCT116, and HCT116 *TP53* ^−/−^ cells with K6 (2, 4, and 8 µM) treated for 24 h. (B) AKT S473D overexpression impaired the suppressive effect of K6 to c‐Myc in CRC cells. The protein expression of c‐Myc was assessed by WB in SW480, HCT116, and HCT116 *TP53* ^−/−^ cells after treated with K6 (8 µM) for 24 h. (C) Overexpression of *c‐Myc* inhibited the killing effect of K6 in CRC cells. The percentages of SW480, HCT116, and HCT116 *TP53* ^−/−^ cells viability were detected by sulforhodamine B (SRB) after treated with K6 (0, 0.5, 1, 2, 4, 6, 8, or 10 µM) for 48 h.

The PI3K/AKT signaling pathway and its downstream effector c‐Myc always promote cell proliferation.^36^, ^37^ As K6 could induce ROS level and ROS has been reported to inhibit PI3K/AKT pathway,^38^ we wonder whether K6 could inhibit the activation of PI3K/AKT. We treated the SW480 cells with 8 µM K6 for 24 h and tested the samples with RNA‐seq. PI3K/AKT pathway was enriched in the results, indicating K6 could inhibit the activation of PI3K/AKT (Figure S4). We observed that K6 reduced the phosphorylation levels of PI3K, AKT, and GSK3β through Western blot analysis (Figure 4A). It has been reported that S9 unphosphorylated GSK3β can directly phosphorylate c‐Myc at T58 site and KLF5 at S303 site, thereby promoting the ubiquitination and proteasomal degradation of c‐Myc and KLF5 through the E3 ubiquitin ligase Fbw7.^36^, ^37^ We validated that K6 reduced the levels of KLF5 and FGF‐BP1 in CRC cells (Figure 4A). These results suggested that K6 inhibits the activity of PI3K/AKT while simultaneously activating GSK3β‐mediated c‐Myc and KLF5 degradation.

Next, we wanted to determine whether K6 inhibits proliferation and induces apoptosis of CRC cells by the AKT signaling pathway. To test this hypothesis, we generated three AKT‐overexpressing cell lines in which the S473 phosphorylation site of AKT was mutated to D to mimic the AKT phosphorylated state. As expected, K6‐induced GSK3β phosphorylation and c‐Myc protein decrease as well as loss of cell viability were partially blocked by AKT overexpression (Figure 4B). Similarly, we overexpressed *c‐Myc* in SW480, HCT116 and HCT116 *TP53* ^−/−^ cells, and found that overexpression of c‐Myc partially reduced the cytotoxicity of K6 in CRC cells (Figure 4C). These results collectively imply that K6 inhibits CRC cell proliferation through inactivate the PI3K/AKT/GSK3β/c‐Myc pathway.

### 2.5 K6 activates PTEN by downregulating c‐Myc/WWP1 expression in CRC cells

**FIGURE 5:**
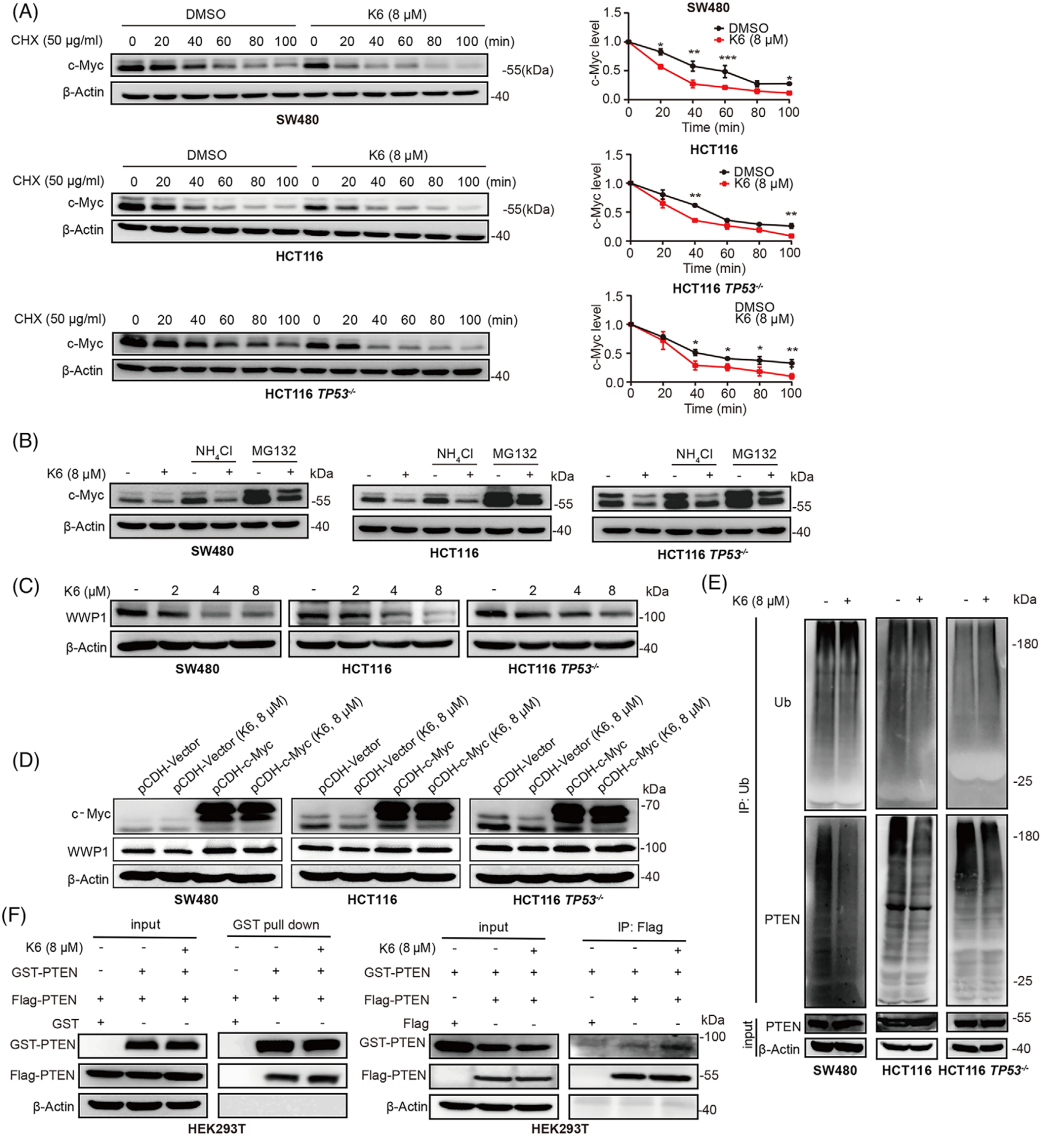
K6 activates phosphatase and tensin homolog (PTEN) by decreasing the expression of the myelocytomatosis (c‐Myc) target gene *WWP1* expression in colorectal cancer (CRC) cells. (A) K6 partially facilitate the degradation of c‐Myc by cyclohexane (CHX) in CRC cells. Following treatment with K6 (8 µM) for 22 h, the cells were exposed to CHX (50 µg/mL) for 0, 20, 40, 60, 80, and 100 min, respectively, c‐Myc protein level was detected by Western blotting (WB). (B) MG132 partially blocked the ability of K6 to downregulate c‐Myc protein levels in CRC cells. After treating with K6 (8 µM) for 20 h, the cells were exposed to MG132 (20 µM) or NH_4_Cl (10 mM) for 4 h, and c‐Myc protein level was detected by WB. (C) K6 downregulated WWP1 protein levels in CRC cells. The protein expression of WWP1 was assessed by WB in SW480, HCT116, and HCT116 *TP53* ^−/−^ cells after treated with K6 (2, 4, and 8 µM) for 24 h. (D) Overexpression of *c‐Myc* weakened the suppressive effect of K6 on WWP1 in CRC cells. The protein expression of WWP1 in SW480, HCT116, and HCT116 *TP53* ^−/−^ cells after treatment with K6 (8 µM) for 24 h was examined by WB. (E) K6 reduced PTEN ubiquitination levels in CRC cells. The ubiquitination level of PTEN in SW480, HCT116, and HCT116 *TP53* ^−/−^ cells after treatment with K6 (8 µM) for 24 h was determined by WB. (F) K6 promoted the dimerization of PTEN. coimmunoprecipitation (Co‐IP) detected the effect of K6 (8 µM) on the dimerization of PTEN in HEK293T cells.

To test whether K6 promotes the c‐Myc proteasomal degradation, we first measured the c‐Myc protein half‐lives by the cycloheximide (CHX) chase assay. As depicted in Figure 5A, K6 notably accelerated the degradation of c‐Myc protein in SW480, HCT116, and HCT116 *TP53* ^−/−^ cells. Furthermore, the K6‐induced reduction in c‐Myc protein levels was partially rescued by the proteasome inhibitor MG132, but not by lysosome inhibitor ammonia chloride (NH_4_Cl) (Figure 5B).

Prior studies have established *WWP1* as the direct downstream target gene of *c‐Myc*, and the WWP1 protein can ubiquitinate PTEN and decrease its cytoplasmic retention and dimerization.^32^ Since K6 downregulated the c‐Myc expression in CRC cells, we examined whether K6 impact the WWP1 expression. In agreement with our speculation, K6 significantly diminished the protein levels of WWP1 in SW480, HCT116, and HCT116 TP53^−/−^ cells (Figure 5C). Overexpression of c‐Myc saved the K6‐induced decline in WWP1 expression (Figure 5D). Then, we demonstrated that K6 indeed reduced the PTEN ubiquitination (Figure 5E). Coimmunoprecipitation (Co‐IP) experiments showed that K6 also promoted PTEN dimerization (Figure 5F). These results indicated that K6 activates PTEN by downregulating c‐Myc/WWP1 expression in CRC cells.

## 3 DISCUSSION

**FIGURE 6:**
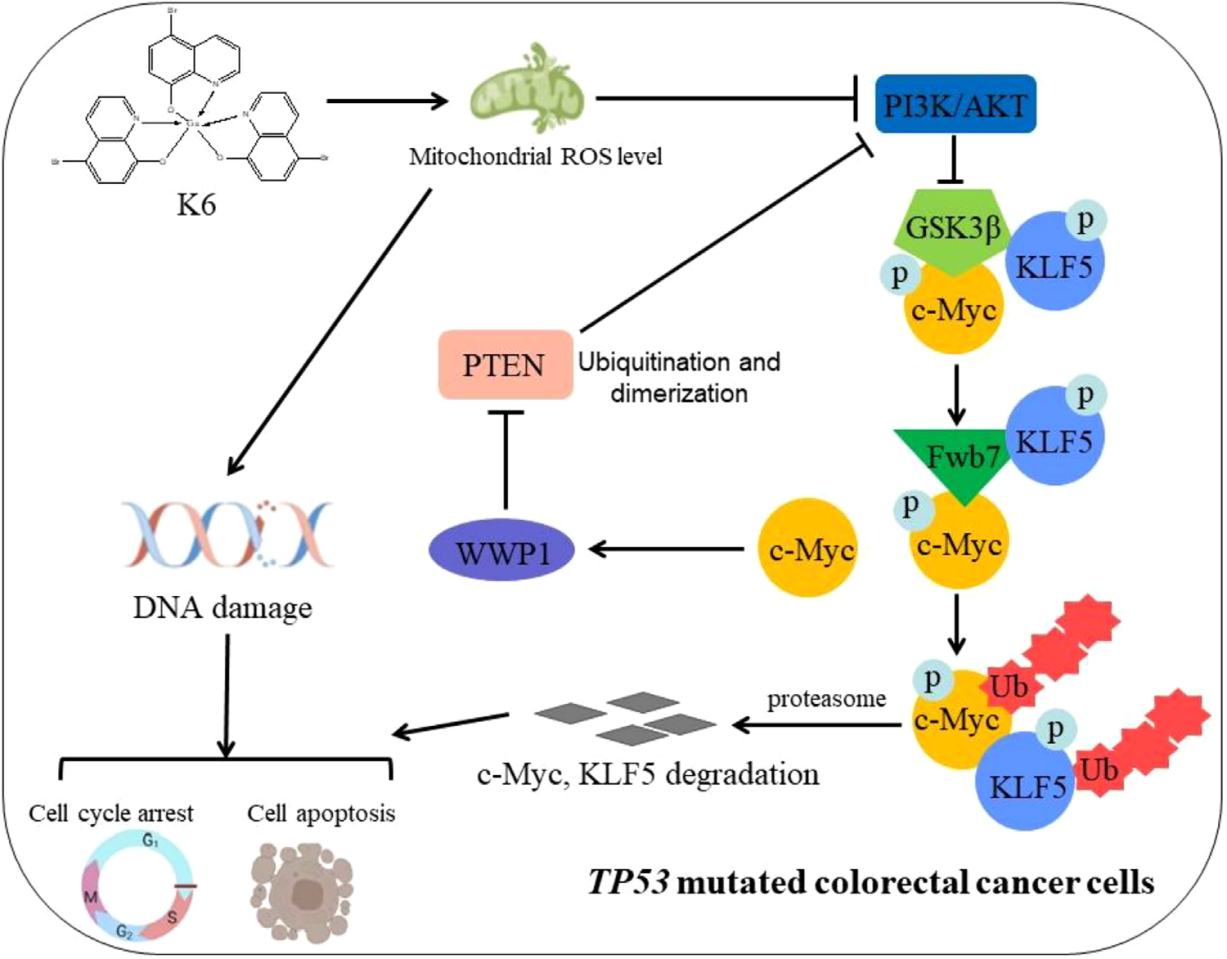
K6 inhibits colorectal cancer (CRC) independent of *TP53* by inducing deoxyribonucleic acid (DNA) damage and apoptosis, and by downregulating myelocytomatosis (c‐Myc) to activate phosphatase and tensin homolog (PTEN).

CRC is a serious malignancy that poses a significant threat to human health and life, and chemotherapy is the main therapeutic strategy. However, the *TP53* mutation confers CRC resistance to platinum drugs, such as L‐OHP. Hence, the development of new drugs for CRC is an urgent need. Here, we identified K6, a gallium complex, as an anti‐CRC compound. On the one hand, K6 increased DNA damage by upregulating the level of intracellular ROS and activating the mitochondrial‐dependent apoptosis pathway independent of *TP53*. Conversely, K6 inhibited the PI3K/AKT signaling and promoted downstream effectors c‐Myc, KLF5 degradation, also the CRC cells proliferation. c‐Myc degradation also decreased WWP1 protein level and activates PTEN (Figure 6). In conclusion, these outcomes implied that K6 is a promising potential therapeutic compound for CRC patients, particularly those harboring *TP53* mutations.

Several studies have shown that various gallium compounds or gallium complexes, such as gallium nitrate, gallium malonate, and KP46, exhibit the capability to suppress the growth of diverse tumor cells and induce cell death. In the clinic, some CRC patients show resistance to platinum drugs because of *TP53* mutations. In this study, we identified that K6 had stronger anticancer activity in a variety of CRC cells than L‐OHP. Most importantly, K6 had a strong killing effect on CRC cells with *TP53* mutations. Thus, K6 may be used as a second‐line therapy to treat CRC patients with L‐OHP resistance.

Previous studies have demonstrated that Ga(III) has the capacity to induce cellular oxidative stress.^39^ Indeed, K6 could increase the level of ROS and cause DNA damage in CRC cells. In addition, K6 also induced the phosphorylation and activation of the DNA damage‐related proteins ATM and CHK1/2. In addition, K6 promoted the cleavage of Caspase‐9 to activate the caspase cascade reaction and to induce apoptosis, suggesting that K6 induced apoptosis of CRC cells through the mitochondrial pathway. Furthermore, we demonstrated that the antioxidant NAC antagonized the DNA damage and apoptosis caused by K6.

Iron is a pivotal component in the function of many proteins that are critical for cell survival and proliferation. For example, iron serves as an essential component in a variety of iron–sulfur‐containing enzymes involved in the citric acid cycle and the mitochondrial electron transport chain. Gallium shares similar chemical properties with iron, which suggests that K6 may disrupt the uptake and utilization of iron by tumor cells, thus affecting mitochondrial function and causing oxidative stress.

Since K6 inhibits CRC independent of *TP53*, we investigated the detailed antitumor mechanisms. We showed that K6 activated another important tumor suppressor, PTEN. K6 significantly reduced the protein level of *c‐Myc*, as well as its downstream target gene *WW*P1. This cascade of events led to the activation of PTEN and subsequent inactivation of the PI3K/AKT signaling pathway. However, whether the transcription level of c‐Myc is inhibited by K6 requires further investigation. In addition, we also found that K6 could ubiquitinate c‐Myc to promote its degradation. Fbw7 is an important E3 ubiquitin ligase known to mediate c‐Myc ubiquitination. Unlike other E3 ubiquitin ligases that directly interact with c‐Myc, Fbw7 recognizes c‐Myc through phosphorylating T58 site, which mediates c‐Myc ubiquitination and then degradation.^40^ Therefore, the T58 site is important for regulating the stability of c‐Myc. Consistently, our study confirmed that K6 could enhance the phosphorylation of c‐Myc at the T85 site in our study. Additionally, previous reports have indicated that the phosphorylation level of c‐Myc at the T58 site is influenced by GSK3β activity. Interestingly, we found that K6 could activate GSK and further promote the ubiquitination level of c‐Myc by reducing PTEN ubiquitination and inhibiting PI3K/AKT activity. Consequently, we speculated that K6 could inhibit the ubiquitination of PTEN by WWP1 through reducing the mRNA level of c‐Myc. Conversely, the PI3K/AKT signaling pathway was inactivated due to the activation of PTEN, causing GSK3β to promote c‐Myc phosphorylation at T58 site, thereby facilitating the degradation of c‐Myc, seemingly forming a positive feedback loop.

WWP1, an intrinsic E3 ubiquitin ligase, can regulate the expression level of substrate proteins, such as Smad2, ErbB4/HER4, KLF5, p63, JunB, and p27, to affect their activity.^41^ Prior research has indicated that *WWP1* is abnormally amplified in prostate cancer and gastric cancer, and may be considered as an oncogene to promote tumor development.^42^, ^43^ However, knockdown *WWP1* promotes cell survival in ERα‐negative breast cancer cells.^44^ These data indicated that *WWP1* has different functions in different tumors. Chen et al. demonstrated that WWP1 exhibits high expression levels in both CRC clinical tissues and cells, which correlates with unfavorable clinical outcomes for patients.^45^ In our study, we found that K6 could downregulate *WWP1* mRNA and protein levels in CRC by inhibiting c‐Myc.

KLF5, a transcription factor, has been recognized to control the cell cycle regulation, apoptosis, migration, and differentiation.^46^ Nandan et al. found that KLF5 was often overexpressed to mediate tumorigenesis in intestinal cells harboring *KRAS* mutations.^47^ Takagi et al. confirmed that in primary CRC, high KLF5 expression promoted cell growth and stem cell‐like characteristics of cancer, thus promoting liver metastasis in patients, which is positively associated with the bleak outlook for patients.^48^ Moreover, Shen et al. proved that ML264, an inhibitor of KLF5, could restore the sensitivity of CRC patient‐derived organoids to L‐OHP by activating the apoptosis pathway.^49^ Consequently, KLF5 emerges as a potential therapeutic objective for the treatment of CRC. We demonstrated that K6 could reduce the protein level of KLF5 and its downstream effector gene FGF‐BP1 by activating GSK‐3β/Fbw7‐mediated KLF5 degradation in our study.

In conclusion, we have identified gallium complex K6 with strong anticancer activity against CRC, providing a theoretical foundation for the potential of K6 as a candidate compound for the therapy of *TP53‐mutated* CRC.

## 4 MATERIALS AND METHODS

### 4.1 Chemicals and antibodies

Gallium complex K6 was synthesized by Professor Mingjin Xie's research group at Yunnan University. Dimethyl sulfoxide (DMSO) (HY‐Y0320), Tween 80 (A100442), polyethylene glycol 300 (202371), NAC (HY‐B0215), and L‐OHP (HY‐17371) were purchased from MCE. FITC Annexin V Apoptosis Detection Kit I (556547) was purchased from BD Biosciences. YF488Click‐iT EdU imaging kit (C6015) was purchased from US Everbright Inc. The antibodies utilized in this investigation comprise anti‐p53 (48818S), anti‐Caspase3/8/9 (9662S/9746S/9508S), anti‐c‐Myc (18583S), anti‐PI3K (17366S), anti‐AKT (4691S), anti‐GSK3β (12456S), anti‐p‐PI3K (T458/T199) (4228S), anti‐p‐AKT (S473) (4060S), and anti‐p‐GSK3β (S9) (5558S) from Cell Signaling Technology. Additionally, anti‐c‐Myc(T58) (sc‐377552) was purchased from Santa Cruz Biotechnology, anti‐WWP1 (ab43791) from Abcam, and anti‐PTEN (60300‐1‐Ig) from Proteintech.

### 4.2 Cell culture and lentivirus infection

Human CRC cell lines with varying TP53 status, including HCT116 TP53WT, RKO TP53WT, SW480 TP53mut, SW620 TP53mut, HCT116 TP53^‒/‒^ and the human renal epithelial cell line HEK293T were cultured in DMEM (Thermo Fisher) supplemented with 5% fetal bovine serum at 37°C with 5% CO_2_. All the cell lines utilized in this investigation were acquired from American Type Culture Collection and demonstrated by short tandem repeat analysis.

The pCDH‐AKT/c‐Myc lentivirus was produced through transfecting HEK293T cells added with packaging plasmids (psPAX2 and pMD2.G). The lentivirus was harvested 48 h after transfection, and used to infect selected cell lines, such as HCT116, SW480, and HCT116 *TP53* ^−/−^. After 48 h of infection, stably infected cells were selected using puromycin (2 µg/mL, InvivoGen, antpr‐1).

### 4.3 Sulforhodamine B assays

Cell viability was assessed through SRB assays. Initially, cells were seeded in 96‐well plates at a density of approximately 5000 cells per well. Upon cell adhesion, medium containing K6 at various concentrations was added, and the cells were then cultured for a duration of 48 h. Afterwards, the medium was replaced with 10% trichloroacetic acid and incubated at room temperature for 30 min. Subsequently, the cells were incubated with 0.4% SRB solution at normal temperature, followed by five washes with 1% acetic acid. At the end, a 10 mM unbuffered Tris base solution was introduced to dissolve SRB, and the absorbance was recorded at 530 nm using a microplate reader (Bio Tek).

### 4.4 5‐Ethynyl‐2‐deoxyuridine assays

For cell proliferation assessment, an EdU imaging kit (US Everbright Inc., C6015) was employed. Cells were initially seeded on coverslips (BD Biosciences) and then treated with either K6 or L‐OHP on the following day. Following the completion of processing steps, cells were incubated with 10 µM EdU for 4 h, following by immobilization, permeabilization, and staining. Subsequently, graphics were captured, and the rate of EdU‐positive cells (%) was determined with the ImageJ software.

### 4.5 Colony formation assays

Briefly, 1000 cells were seeded in a six‐well plate, cultured overnight and exposed to concentrations gradient of K6. The culture was maintained until visible colonies developed in the control group, typically requiring around 2 weeks. At this point, cells were immobilized with 4% paraformaldehyde and stained with 0.5% crystal violet. After rinsing to eliminate excess dye, colony quantification was conducted using a microscope.

### 4.6 Western blotting

Cells were lysed in RIPA buffer supplemented with protease inhibitor for 30 min on ice to obtain protein samples, and further determine the protein concentration. The protein samples underwent thermal denaturation followed by separation through sodium dodecyl sulfate polyacrylamide gel electrophoresis (SDS‐PAGE) gels and subsequent transfer onto polyvinylidene difluoride (PVDF) membranes, then blocked using 5% nonfat milk. After blocking, the membranes were incubated with specific primary antibodies overnight at 4°C, then treated with the corresponding secondary antibodies next day. Finally, the target protein signals were measured using enhanced chemiluminescence (ECL) detection reagents (US, P0018A) with the ImageQuant LAS 4000 System.

### 4.7 Quantitative real‐time PCR

The mRNA from treated cells was extracted by TRIzol method initially. Then, obtaining the cDNA through reverse transcription assay and quantifying the genes’ expression by using SYBR Green Select Master Mix (Applied Biosystems, 4472908). The primer sequences were designed and then synthesized. The primer sequences are listed in Table S1.

### 4.8 Measurement of reactive oxygen species

Intracellular ROS were assessed through the 2′,7′‐dichlorofluorescin diacetate (DCFH‐DA) reagent (Beyotime, S0033). In brief, cells were cultured with 10 µM DCFH‐DA in a dark environment. Subsequently, the cells were washed by phosphate‐buffered saline (PBS) twice to eliminate redundant probe, and the percentage of combinative cells in 20,000 was analyzed by flow cytometry (BD Biosciences). Data analysis was performed using FlowJo software.

### 4.9 Apoptosis analysis and cell cycle

To evaluate the impact of K6 treatments on apoptosis, Annexin‐V and propidium iodide (PI)a staining assays (Apoptosis Detection Kit, BD Biosciences, 556547) were conducted. In summary, treated cells with K6 for 24 h, followed by trypsinization, collection, and staining with Annexin V and PI, and flow cytometry was used to assess the percentage of apoptotic cells. For cell cycle analysis, cells were treated with K‐6 for 24 h, harvested, and fixed overnight at 4°C in 75% ethanol. Cells were centrifuged, washed with PBS, and incubated with 100 µL of 0.6% NP‐40 solution containing the DNA fluorescent dyes PI and RNaseA for 30 min at 37°C in the dark circumstances. The cell cycle distribution was assessed by flow cytometry. The data were analyzed using FlowJo software.

### 4.10 Immunofluorescence

Following treatment with K6 or L‐OHP for the indicated times, cells were immobilized with 4% paraformaldehyde (PFA), permeabilized in 0.5% Triton X‐100, blocked with 5% bovine serum albumin (BSA) and then incubated with the γH2Ax antibodies at 4°C overnight. The next day, cells were incubated with fluorescein‐signed Alexa Fluor 488‐labeled secondary antibody for 1 h. 4', 6‐diamidino‐2‐phenylindole (DAPI) was utilized to stain the nuclei. Fluorescence images were acquired with a fluorescence microscope (Carl Zeiss, Axio Imager A2, Oberkochen, GER).

### 4.11 Coimmunoprecipitation and GST pull‐down

c‐Myc was cloned into the pCDH‐3×Flag or pEBG‐GST vector. First, overexpressing Flag‐c‐Myc in HEK293T cells through transfecting plasmids by PEI reagent (Invitrogen, 23966). Transfected HEK293T cells were lysed in IP lysis buffer added with 1% protease inhibitor cocktail (Bimake, B14001). After centrifugation, the supernatants were removed to new tubes and rotated with Flag‐M2 beads (Sigma–Aldrich, F3165) for 2 h. For GST pull‐down experiment, overexpressing GST‐c‐Myc by transfected pEBG‐c‐Myc plasmids into HEK293T cells. Collected cell lysed buffer, centrifugated and collected supernatants, rotated with glutathione Sepharose 4B beads (GE Healthcare) for 2 h. The beads from both Co‐IP and GST pull‐down were washed at least five times by IP lysis buffer before being separated and immunoblotted.

### 4.12 Cycloheximide chase assays

After treated with K6 for 24 h, the cells were exposed with MG132 (20 µM) or NH_4_Cl (10 mM) for 4 h, and then incubated with 50 µg/mL CHX at designed time, ranging from 0 to 100 min. Subsequently, proteins were extracted and conducted to Western blotting. The expression level of c‐Myc protein was determined by the ImageJ software.

### 4.13 Ubiquitination assays

After treated with K6 for 24 h, the cells were lysed in SDS lysis buffer (1.5% SDS, 50 mM Tris–Cl, pH 6.8) and then heated at 98°C for 10 min. Then, slight protein lysate was kept as input samples, the great mass of lysate was diluted 10‐fold with cold BSA buffer (0.5% NP‐40, 0.5% BSA, 180 mM NaCl, and 50 mM Tris‐Cl, pH 6.8) and mixed with the ubiquitin antibody overnight. The following day, a mixture of protein lysate and antibody was gently rotated with Protein A/G‐plus Agarose (Santa Cruz, sc‐2003) for 6 h. The immunoprecipitants were washed at least five times with BSA buffer to isolated the uncombined proteins, the immunoprecipitated proteins were detected by Western blot assay.

### 4.14 Animal experiments

We purchased nude mice from Hunan SJA Laboratory and allowed a 1‐week acclimatization period. The experimental protocols for animal studies got permission from the animal ethics committee of the Kunming Institute of Zoology, CAS. SW480 cells were implanted subcutaneously at the right anterior flanks of the mice, approximately 3 × 10^6^ cells each point. Until the tumors had grown to about 50−70 mm^3^ in volume, tumor‐bearing mice were randomly divided into four groups (*n* = 6), and then received equal amounts of solvent, DMSO, K6 (5 mg/kg), K6 (10 mg/kg), or L‐OHP (10 mg/kg) via intraperitoneal injection every 2 days for a duration of 14 days. The tumor sizes of the mice and their body weights were assessed and documented every 3 days. The tumor volume was calculated following the formula (π × length × width^2^)/6. At the end of the animal experiment, all tumor‐bearing mice were sacrificed, and the tumors were harvested, weighed, and analyzed.

### 4.15 Statistical analysis

All experiments were conducted at least three times. The data analysis involved was performed using GraphPad Prism 9.0.0, and the statistical results were all presented as the mean ± SD. Student's *t*‐test was utilized to assess the significance between control group and treated group. Multiple comparisons were analyzed by two‐way analysis of variance. In the statistical representation, ns, not significant, ^**^ *p *< 0.01, significant difference, and ^***^ *p *< 0.001, particularly significant difference.

## AUTHOR CONTRIBUTIONS

C.C., J.H., and W.L. conceived and designed the experiments. W.L., C.Y., and Z.C. performed most experiments and analyzed data. X.Q. and Y.Z. assisted in conducting animal experiments. M.X., Y.W., and S.Z. synthesized all gallium complexes. W.L. and C.C. drafted and edited the manuscript. All authors have reviewed and approved the final manuscript.

## CONFLICT OF INTEREST STATEMENT

The authors declare they have no conflicts of interest.

## ETHICS STATEMENT

The animal experiments were approved by the Kunming Institute of Zoology (IACUC‐PA‐2022‐03‐047).

## Supporting information

Supporting Information

## Data Availability

The authors confirm that the data from this study are available, including the figures and Supporting Information.

## References

[1] Thanikachalam K , Khan G . Colorectal cancer and nutrition. Nutrients. 2019;11(1):164. 10.3390/nu11010164 PMC6357054 30646512

[2] Elias D , Lefevre JH , Chevalier J , et al. Complete cytoreductive surgery plus intraperitoneal chemohyperthermia with oxaliplatin for peritoneal carcinomatosis of colorectal origin. J Clin Oncol. 2009;27(5):681‐685. 19103728 10.1200/JCO.2008.19.7160

[3] Nagasaka T , Mishima H , Sawaki A , et al. Protocol of a randomised phase III clinical trial of sequential capecitabine or 5‐fluorouracil plus bevacizumab (Cape/5‐FU‐Bmab) to capecitabine or 5‐fluorouracil plus oxaliplatin plus bevacizumab (CapeOX/mFOLFOX6‐Bmab) versus combination CapeOX/mFOLFOX6‐Bmab in advanced colorectal cancer: the C‐cubed (C3) study. BMJ Open. 2016;6(6):e011454. 10.1136/bmjopen-2016-011454 PMC4893850 27256093

[4] Suh YA , Post SM , Elizondo‐Fraire AC , et al. Multiple stress signals activate mutant p53 in vivo. Cancer Res. 2011;71(23):7168‐7175. 21983037 10.1158/0008-5472.CAN-11-0459 PMC3320147

[5] Michel M , Kaps L , Maderer A , Galle PR , Moehler M . The role of p53 dysfunction in colorectal cancer and its implication for therapy. Cancers (Basel). 2021;13(10):2296. 10.3390/cancers13102296 PMC8150459 34064974

[6] Olivier M , Hollstein M , Hainaut P . TP53 mutations in human cancers: origins, consequences, and clinical use. Cold Spring Harb Perspect Biol. 2010;2(1):a001008. 20182602 10.1101/cshperspect.a001008 PMC2827900

[7] Farrell NP , Gorle AK , Peterson EJ , Berners‐Price SJ . Metalloglycomics. Met Ions Life Sci. 2018;18：/books/9783110470734/9783110470734‐9783110470007/9797831104707 10.1515/9783110470734-010 29394023

[8] Hart MM , Adamson RH . Antitumor activity and toxicity of salts of inorganic group I11a metals: aluminum, gallium, indium, and thallium. Proc Natl Acad Sci U S A. 1971;68:1623‐1626. 5283954 10.1073/pnas.68.7.1623 PMC389254

[9] Valiahdi SM , Heffeter P , Jakupec MA , et al. The gallium complex KP46 exerts strong activity against primary explanted melanoma cells and induces apoptosis in melanoma cell lines. Melanoma Res. 2009;19(5):283‐293. 19584767 10.1097/CMR.0b013e32832b272d PMC3371751

[10] Chua M‐S , Bernstein LR , Li R , So SKS . Gallium maltolate is a promising chemotherapeutic agent for the treatment of hepatocellular carcinoma. Anticancer Res. 2006;26:1739‐1744. 16827101

[11] Chitambar CR . Gallium‐containing anticancer compounds. Future Med Chem. 2012;4(10):1257‐1272. 22800370 10.4155/fmc.12.69 PMC3574811

[12] Chitambar CR , Antholine WE . Iron‐targeting antitumor activity of gallium compounds and novel insights into triapine((R))‐metal complexes. Antioxid Redox Signal. 2013;18(8):956‐972. 22900955 10.1089/ars.2012.4880 PMC3557436

[13] Er E , Oliver L , Cartron PF , Juin P , Manon S , Vallette FM . Mitochondria as the target of the pro‐apoptotic protein Bax. Biochim Biophys Acta. 2006;1757(9‐10):1301‐1311. 16836974 10.1016/j.bbabio.2006.05.032

[14] Dang CV . MYC on the path to cancer. Cell. 2012;149(1):22‐35. 22464321 10.1016/j.cell.2012.03.003 PMC3345192

[15] Walz S , Lorenzin F , Morton J , et al. Activation and repression by oncogenic MYC shape tumour‐specific gene expression profiles. Nature. 2014;511(7510):483‐487. 25043018 10.1038/nature13473 PMC6879323

[16] Porter JR , Fisher BE , Baranello L , et al. Global inhibition with specific activation: how p53 and MYC redistribute the transcriptome in the DNA double‐strand break response. Mol Cell. 2017;67(6):1013‐1025. 28867293 10.1016/j.molcel.2017.07.028 PMC5657607

[17] Ho JS , Ma W , Mao DY , Benchimol S . p53‐Dependent transcriptional repression of c‐Myc is required for G1 cell cycle arrest. Mol Cell Biol. 2005;25(17):7423‐7431. 16107691 10.1128/MCB.25.17.7423-7431.2005 PMC1190302

[18] Choi SH , Wright JB , Gerber SA , Cole MD . Myc protein is stabilized by suppression of a novel E3 ligase complex in cancer cells. Genes Dev. 2010;24(12):1236‐1241. 20551172 10.1101/gad.1920310 PMC2885659

[19] Sears R , Nuckolls F , Haura E , Taya Y , Tamai K , Nevins JR . Multiple Ras‐dependent phosphorylation pathways regulate Myc protein stability. Genes Dev. 2000;14(19):2501‐2514. 11018017 10.1101/gad.836800 PMC316970

[20] Dai MS , Jin Y , Gallegos JR , Lu H . Balance of Yin and Yang: ubiquitylation‐mediated regulation of p53 and c‐Myc. Neoplasia. 2006;8(8):630‐644. 16925946 10.1593/neo.06334 PMC1601943

[21] Liu R , Chen Y , Liu G , et al. PI3K/AKT pathway as a key link modulates the multidrug resistance of cancers. Cell Death Dis. 2020;11(9):797. 32973135 10.1038/s41419-020-02998-6 PMC7515865

[22] Song MS , Carracedo A , Salmena L , et al. Nuclear PTEN regulates the APC‐CDH1 tumor‐suppressive complex in a phosphatase‐independent manner. Cell. 2011;144(2):187‐199. 21241890 10.1016/j.cell.2010.12.020 PMC3249980

[23] Planchon SM , Waite KA , Eng C . The nuclear affairs of PTEN. J Cell Sci. 2008;121(pt 3):249‐253. 18216329 10.1242/jcs.022459

[24] Lee YR , Chen M , Pandolfi PP . The functions and regulation of the PTEN tumour suppressor: new modes and prospects. Nat Rev Mol Cell Biol. 2018;19(9):547‐562. 29858604 10.1038/s41580-018-0015-0

[25] Shen WH , Balajee AS , Wang J , et al. Essential role for nuclear PTEN in maintaining chromosomal integrity. Cell. 2007;128(1):157‐170. 17218262 10.1016/j.cell.2006.11.042

[26] Xie P , Peng Z , Chen Y , et al. Neddylation of PTEN regulates its nuclear import and promotes tumor development. Cell Res. 2021;31(3):291‐311. 33299139 10.1038/s41422-020-00443-z PMC8027835

[27] Hong SW , Moon JH , Kim JS , et al. p34 is a novel regulator of the oncogenic behavior of NEDD4‐1 and PTEN. Cell Death Differ. 2014;21(1):146‐160. 24141722 10.1038/cdd.2013.141 PMC3857621

[28] Ma L , Yao N , Chen P , Zhuang Z . TRIM27 promotes the development of esophagus cancer via regulating PTEN/AKT signaling pathway. Cancer Cell Int. 2019;19:283. 31719796 10.1186/s12935-019-0998-4 PMC6839104

[29] Van Themsche C , Leblanc V , Parent S , Asselin E . X‐linked inhibitor of apoptosis protein (XIAP) regulates PTEN ubiquitination, content, and compartmentalization. J Biol Chem. 2009;284(31):20462‐20466. 19473982 10.1074/jbc.C109.009522 PMC2742810

[30] Maddika S , Kavela S , Rani N , et al. WWP2 is an E3 ubiquitin ligase for PTEN. Nat Cell Biol. 2011;13(6):728‐733. 21532586 10.1038/ncb2240 PMC3926303

[31] Sun G , Ye H , Wang X , et al. Identification of novel autoantibodies based on the protein chip encoded by cancer‐driving genes in detection of esophageal squamous cell carcinoma. Oncoimmunology. 2020;9(1):1814515. 33457096 10.1080/2162402X.2020.1814515 PMC7781740

[32] Lee YR , Chen M , Lee JD , et al. Reactivation of PTEN tumor suppressor for cancer treatment through inhibition of a MYC‐WWP1 inhibitory pathway. Science. 2019;364(6441):eaau0159. 10.1126/science.aau0159 PMC7081834 31097636

[33] Zhou S‐H , Wang R‐D , Wu T‐T , et al. Long rod‐shaped gallium composite material: self‐separating material aggregation induced enhancement of ROS for photothermal/photodynamic therapy of HCT116 cells. Eur J Med Chem. 2023;262:115892. 10.1016/j.ejmech.2023.115892 39491428

[34] Zhou S‐H , Liao W‐H , Yang Y , et al. 8‐Hydroxyquinoline gallium(III) complex with high antineoplastic efficacy for treating colon cancer via multiple mechanisms. ACS Omega. 2023;8:6945‐6958. 36844596 10.1021/acsomega.2c07742 PMC9948165

[35] Rodrigues NR , Rowan A , Smith ME , et al. p53 mutations in colorectal cancer. Proc Natl Acad Sci U S A. 1990;87:7555‐7559. 1699228 10.1073/pnas.87.19.7555 PMC54786

[36] Wang X , Qiu T , Wu Y , et al. Arginine methyltransferase PRMT5 methylates and stabilizes KLF5 via decreasing its phosphorylation and ubiquitination to promote basal‐like breast cancer. Cell Death Differ. 2021;28(10):2931‐2945. 33972717 10.1038/s41418-021-00793-0 PMC8481478

[37] Zhao D , Zheng HQ , Zhou Z , Chen C . The Fbw7 tumor suppressor targets KLF5 for ubiquitin‐mediated degradation and suppresses breast cell proliferation. Cancer Res. 2010;70(11):4728‐4738. 20484041 10.1158/0008-5472.CAN-10-0040

[38] Chen K , Li Y , Zhang X , Ullah R , Tong J , Shen Y . The role of the PI3K/AKT signalling pathway in the corneal epithelium: recent updates. Cell Death Dis. 2022;13(5):513. 35641491 10.1038/s41419-022-04963-x PMC9156734

[39] Yang M , Chitambar CR . Role of oxidative stress in the induction of metallothionein‐2A and heme oxygenase‐1 gene expression by the antineoplastic agent gallium nitrate in human lymphoma cells. Free Radic Biol Med. 2008;45(6):763‐772. 18586083 10.1016/j.freeradbiomed.2008.05.031 PMC2610863

[40] Welcker M , Wang B , Rusnac DV , et al. Two diphosphorylated degrons control c‐Myc degradation by the Fbw7 tumor suppressor.pdf. Sci Adv. 2022;8:eabl7872. 35089787 10.1126/sciadv.abl7872 PMC8797792

[41] Zhi X , Chen C . WWP1: a versatile ubiquitin E3 ligase in signaling and diseases. Cell Mol Life Sci. 2012;69(9):1425‐1434. 22051607 10.1007/s00018-011-0871-7 PMC11114891

[42] Goto Y , Kojima S , Kurozumi A , et al. Regulation of E3 ubiquitin ligase‐1 (WWP1) by microRNA‐452 inhibits cancer cell migration and invasion in prostate cancer. Br J Cancer. 2016;114(10):1135‐1144. 27070713 10.1038/bjc.2016.95 PMC4865980

[43] Li Q , Li Z , Wei S , et al. Overexpression of miR‐584‐5p inhibits proliferation and induces apoptosis by targeting WW domain‐containing E3 ubiquitin protein ligase 1 in gastric cancer. J Exp Clin Cancer Res. 2017;36(1):59. 28431583 10.1186/s13046-017-0532-2 PMC5401563

[44] Li Y , Zhou Z , Chen C . WW domain‐containing E3 ubiquitin protein ligase 1 targets p63 transcription factor for ubiquitin‐mediated proteasomal degradation and regulates apoptosis. Cell Death Differ. 2008;15(12):1941‐1951. 18806757 10.1038/cdd.2008.134

[45] Chen JJ , Zhang W . High expression of WWP1 predicts poor prognosis and associates with tumor progression in human colorectal cancer. Am J Cancer Res. 2018;8:256‐265. 29511596 PMC5835693

[46] Jiang D , Qiu T , Peng J , et al. YB‐1 is a positive regulator of KLF5 transcription factor in basal‐like breast cancer. Cell Death Differ. 2022;29(6):1283‐1295. 35022570 10.1038/s41418-021-00920-x PMC9177637

[47] Nandan MO , McConnell BB , Ghaleb AM , et al. Kruppel‐like factor 5 mediates cellular transformation during oncogenic KRAS‐induced intestinal tumorigenesis. Gastroenterology. 2008;134(1):120‐130. 18054006 10.1053/j.gastro.2007.10.023 PMC2194652

[48] Takagi Y , Sakai N , Yoshitomi H , et al. High expression of Kruppel‐like factor 5 is associated with poor prognosis in patients with colorectal cancer. Cancer Sci. 2020;111(6):2078‐2092. 32279400 10.1111/cas.14411 PMC7293098

[49] Shen X , Zhang Y , Xu Z , et al. KLF5 inhibition overcomes oxaliplatin resistance in patient‐derived colorectal cancer organoids by restoring apoptotic response. Cell Death Dis. 2022;13(4):303. 35379798 10.1038/s41419-022-04773-1 PMC8980070

