# Correction to “Gallium Complex K6 Inhibits Colorectal Cancer by Increasing ROS Levels to Induce DNA Damage and Enhance Phosphatase and Tensin Homolog Activity”

**DOI:** 10.1002/mco2.70527

**Published:** 2026-01-04

Li W, Yang C, Cheng Z, et al., “Gallium complex K6 inhibits colorectal cancer by increasing ROS levels to induce DNA damage and enhance phosphatase and tensin homolog activity.” *MedComm*. (2024);5:e665. doi: 10.1002/mco2.665

In the process of checking the raw data [1], the authors noticed an inadvertent mistake occurring in Figure 3B that needed to be corrected after the online publication of the article. In paragraph 3 of the “Results” section, the image data for SW620 cells following treatment with L‐OHP (8 µM) orK6 (8 µM) for 2 h in Figure 3B was incorrect and has been replaced with the correct images. The corrected result is shown below.

**FIGURE 3:**
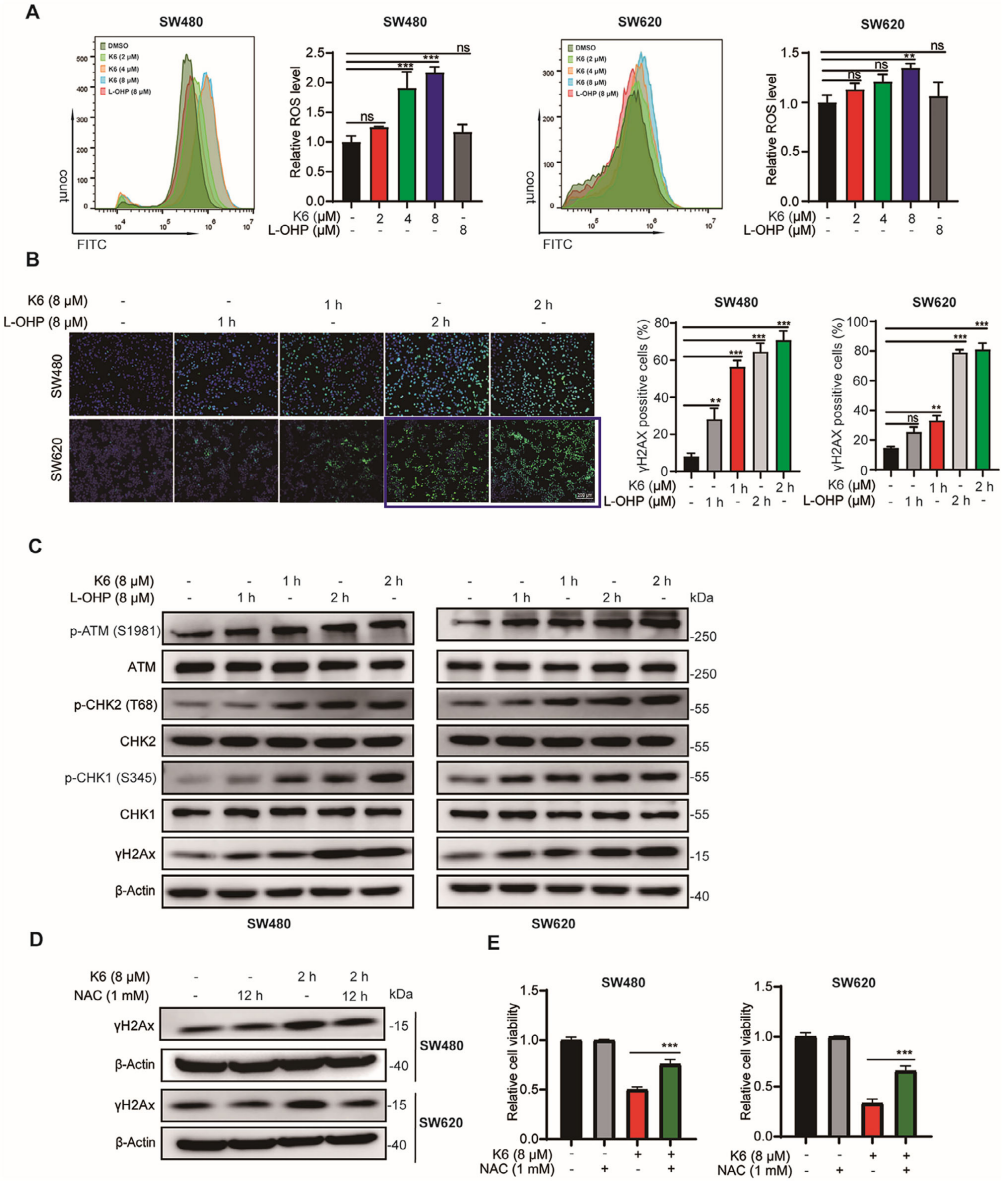
K6 upregulated reactive oxygen species (ROS) levels to induce deoxyribonucleic acid (DNA) damage in SW480 and SW620 cell lines. (A) K6 upregulated ROS levels in SW480 and SW620 cells. The ROS levels of SW480 and SW620 cells after treatment with K6 (2, 4, and 8 µM) or oxaliplatin (L‐OHP) (8 µM) for 6 h were assessed by flow cytometry. (B) K6 induced the rapid accumulation of γH2Ax in SW480 and SW620 cells. The γH2Ax accumulation levels in SW480 and SW620 cells after treatment with K6 (8 µM) or L‐OHP (8 µM) for 1 and 2 h were assessed by immunofluorescence. (C) K6 upregulated the phosphorylation of DNA damage‐related proteins in SW480 and SW620 cells. The phosphorylation levels of ATM, CHK1, CHK2, and H2Ax proteins in SW480 and SW620 cells after treatment with K6 (8 µM) or L‐OHP (8 µM) for 1 and 2 h were detected by Western blotting (WB). (D) N‐acetyl‐L‐cysteine (NAC) antagonized K6‐induced upregulation of γH2Ax protein levels. Following pretreatment of SW480 and SW620 cells with NAC (1 mM) for 12 h, cells were treated with K6 (8 µM) for 2 h, and γH2Ax protein levels were assessed by WB. (E) NAC inhibited the ability of K6 to downregulate the activity of SW480 and SW620 cells. SW480 and SW620 cells were treated with NAC (1 mM) or K6 (8 µM) alone or a combination of both for 48 h, and the cell viability was measured using sulforhodamine B (SRB).

The authors apologize for this error and declare that this correction does not affect the description, interpretation, or conclusions.

1. Li W, Yang C, Cheng Z, et al., “Gallium complex K6 inhibits colorectal cancer by increasing ROS levels to induce DNA damage and enhance phosphatase and tensin homolog activity.” MedComm (2020). 2024 Jul 24;5(8):e665. https://doi.org/10.1002/mco2.665.

