# Supplementary material for: Gallium complex K6 inhibits colorectal cancer by increasing ROS levels to induce DNA damage and enhance phosphatase and tensin homolog activity
Source: MedComm (2020). 2024 Jul 24;5(8):e665. doi: 10.1002/mco2.665 (PMC11266899; doi:10.1002/mco2.665)
Supplement: Supplementary file 1 — Supporting Information [file MCO2-5-e665-s001.docx]

**Gallium** **complex K6 inhibits colorectal cancer by increasing ROS levels to induce DNA damage and enhance Phosphatase and Tensin Homolog activity**

Wei Li ^1, 2, #^, Chuanyu Yang ^1, #^, Zhuo Cheng ^1, 2, #^, Yuanyuan Wu ^3, #^, Sihan Zhou ^3^, Xiaowei Qi ^4^, Yi Zhang ^4^, Jinhui Hu ^5,^ *, Mingjin Xie ^3,^ *, Ceshi Chen ^6, 7,^ ^1,^ *

1 Key Laboratory of Animal Models and Human Disease Mechanisms Yunnan Province, KIZ-CUHK Joint Laboratory of Bioresources and Molecular Research in Common Diseases, Kunming Institute of Zoology, Kunming, 650201, China

2 Kunming College of Life Sciences, University of Chinese Academy Sciences, Kunming, 650204, China

3 School of Chemical Science and Technology, Yunnan University, Kunming, 650091, Yunnan, China

4 Department of Breast and Thyroid Surgery, Southwest Hospital, the First Affiliated Hospital of the Army Military Medical University, Chongqing 400038, China

5 The First Hospital of Hunan University of Chinese Medicine, Changsha, Hunan, 410021，China

6 Academy of Biomedical Engineering, Kunming Medical University, Kunming 650500, China

7 The Third Affiliated Hospital, Kunming Medical University, Kunming 650118, China

^#^ These authors contributed equally

*Corresponding authors, E-mail addresses: chenc@kmmu.edu.cn (C. Chen). mjxie@ynu.edu.cn (M. Xie), [hujinhui173@163.com](mailto:hujinhui173@163.com) (J. Hu)

Figure 1 K6 inhibited the growth of colorectal cancer (CRC) cells

Figure 2 K6 inhibited the proliferation and induced apoptosis in SW480 and SW620 cell lines

Figure 3 K6 upregulated Reactive Oxygen Species (ROS) levels to induce DNA damage in SW480 and SW620 cell lines

Figure 4 K6 functions partially through inactivate the PI3K/AKT/GSK3β/c-Myc pathway

Figure 5 K6 activates PTEN by decreasing the expression of the c-Myc target gene *WWP1* expression in CRC cells

Figure 6 K6 inhibits CRC independent of *TP53* by inducing DNA damage and apoptosis, and by downregulating c-Myc to activate PTEN.

FigureS1 K6 did not have obvious hepatorenal toxicity and decreased the levels of Ki-67, c-Myc and PTEN in vivo.

FigureS2 K6 can inhibit the tumor growth and significantly prolong the survival curve of MC38 tumor-bearing mice.

FigureS3 K6 inhibited the cell proliferation and induced the apoptosis of SW480 and SW620 cells

FigureS4 K6 affects the PI3K-AKT pathway.

Table S1 Primers sequences for qRT-PCR

Table S2 Routine blood of mice

**Supplementary Figures**

**FigureS1 K6 did not have obvious hepatorenal toxicity and decreased the levels of Ki-67, c-Myc and PTEN in vivo.**

SW480 cells were subcutaneously injected into nude mice (3×10^6^/point, n=6). When the tumor volume reached to 50-75 mm^3^, mice were administered K6 or L-OH by intraperitoneal injection.


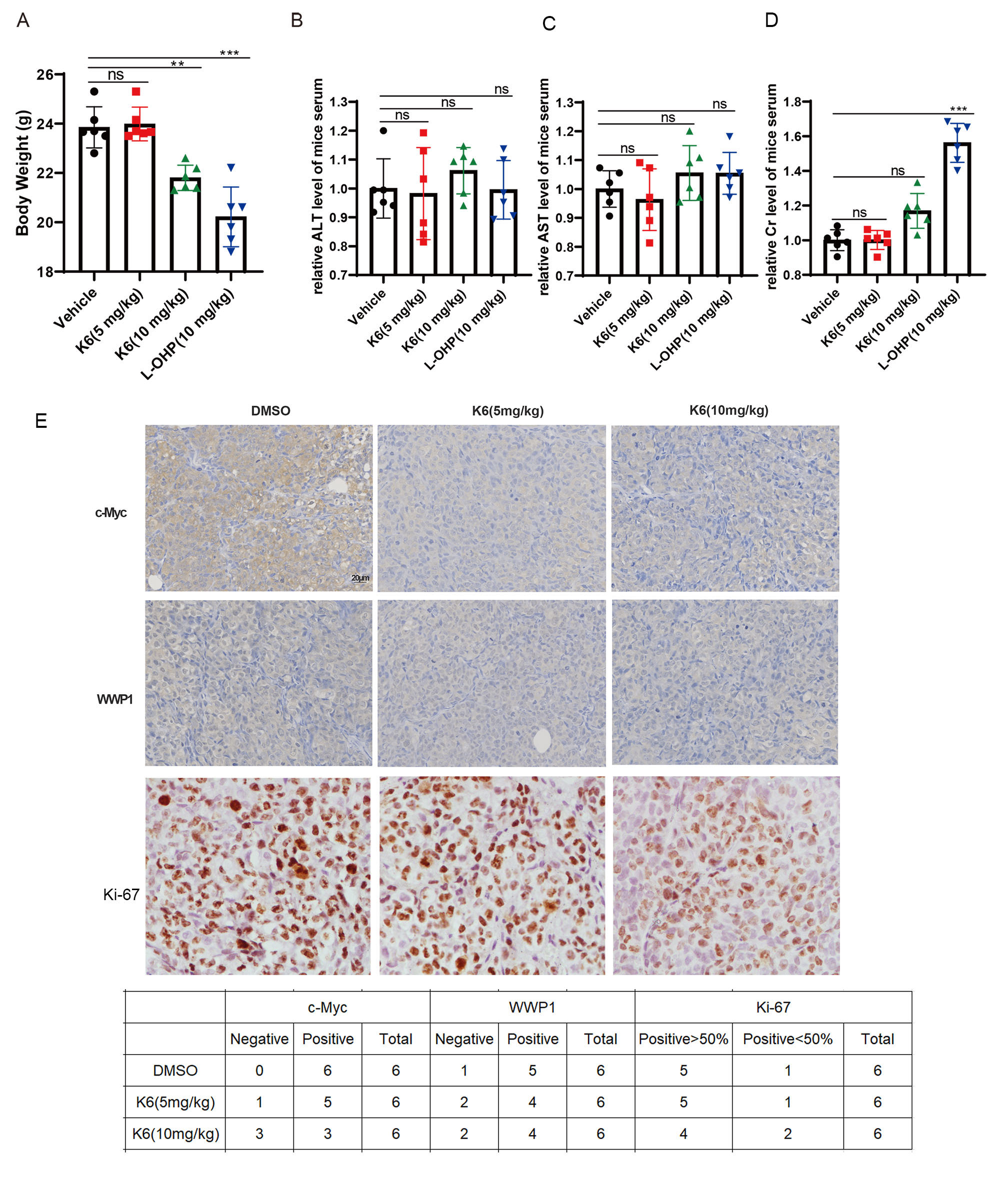


A: The body weight of experimental mice was weighed. K6 did not affect body weight.

B-C: Serum alanine aminotransferase/aspartate aminotransferase (ALT/AST) were detected to reflect the degree liver injury; K6 did not affect serum levels of aminotransferase (AST/ALT).

D: Serum creatinine (Cr) was detected to reflect the degree of kidney injury. K6 did not affect serum levels of creatinine.

E: Immunohistochemical staining was used for Ki-67, c-Myc and PTEN staining in tumor tissue from SW480 tumor-bearing mice and performed statistical analysis.

**FigureS2 K6 can inhibit the tumor growth and significantly prolong the survival curve of MC38 tumor-bearing mice.**


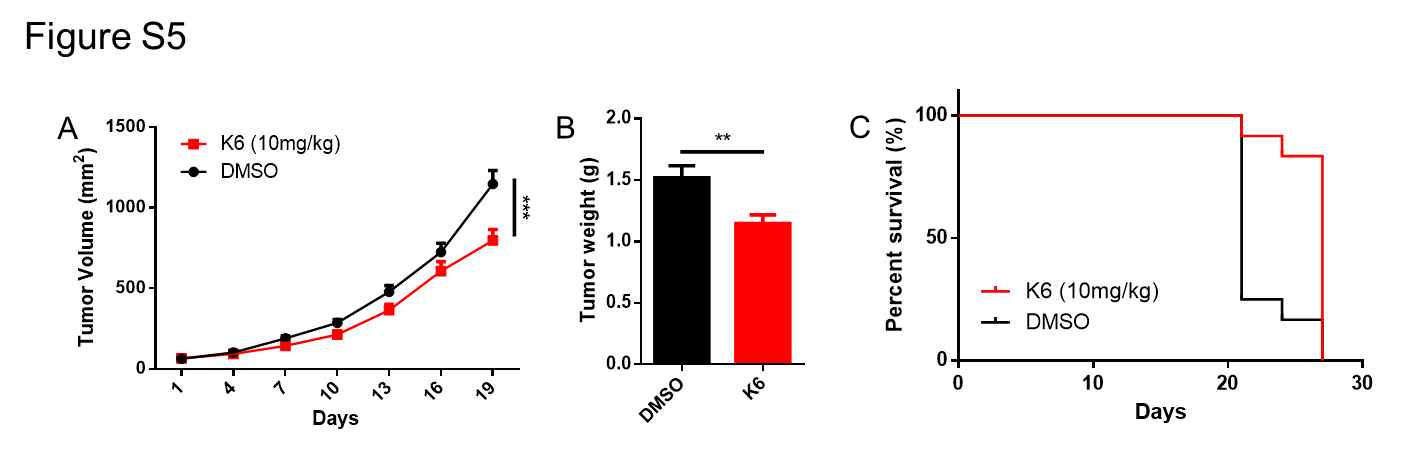


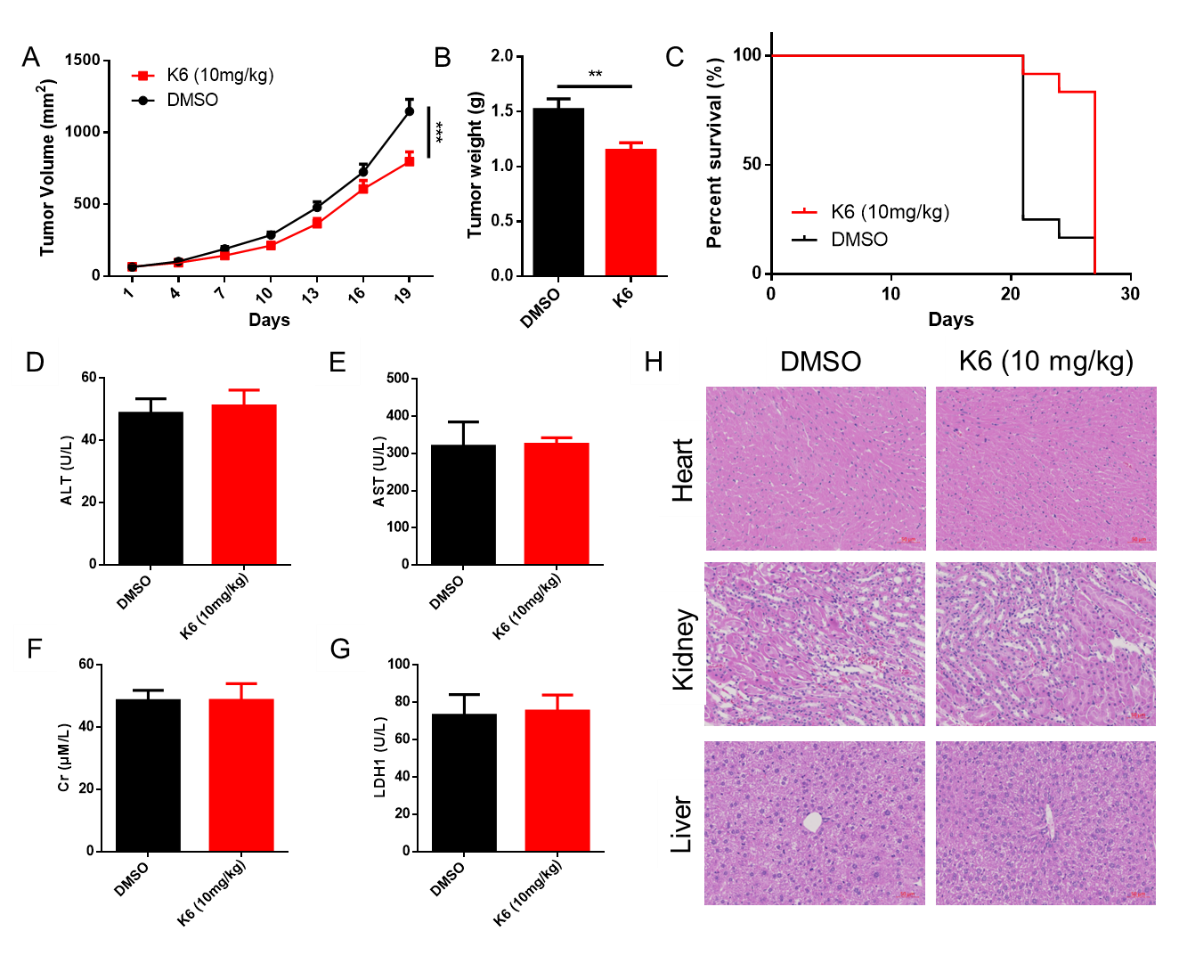


A-B: MC38 cells were subcutaneously injected into nude mice (3×106/point, n=6). When the tumor volume reached to 50-75 mm3, mice were administered K6 by intraperitoneal injection. The tumor volume and weight were measured throughout the 3-week treatment period.

C: Survival curves were detected in MC38 tumor-bearing mice.

D-G: Serum ALT/AST/Cr/LDH1 were detected in MC38 tumor-bearing mice.

H: The heart, kidney and liver from MC38 tumor-bearing mice were stained with HE.

**FigureS3 K6 inhibited the cell proliferation and induced the apoptosis of SW480 and SW620 cells**


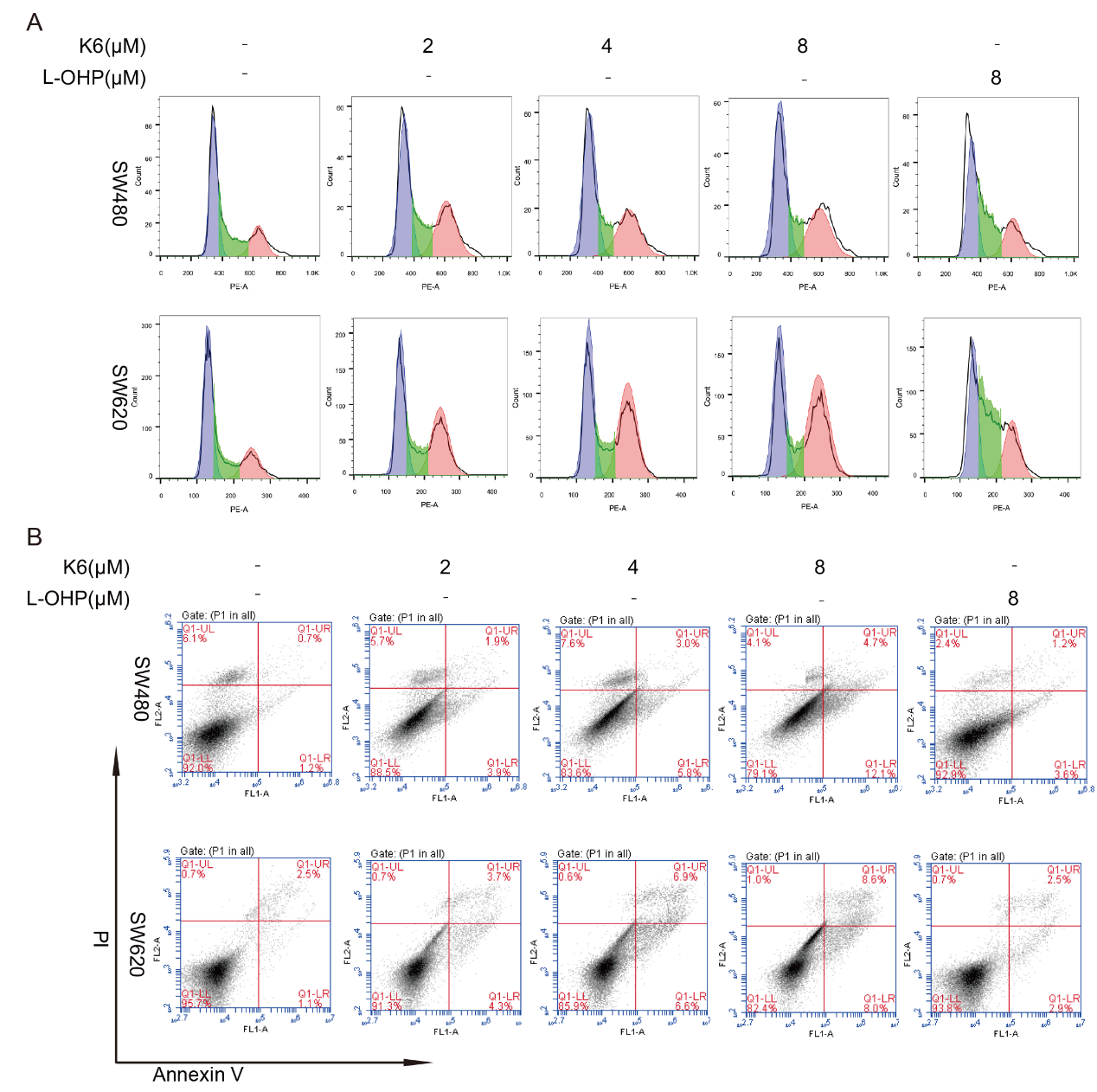


A: K6 arrested the cell cycle of SW480 and SW620 cells in G2/M phase. The cell cycle of SW480 and SW620 cells after treatment with K6 (2, 4, 8 μM), or L-OHP (8 μM) for 24 h was detected by flow cytometry.

B: K6 induced apoptosis of SW480 and SW620 cells. The cell apoptosis of SW480 and SW620 cells after treatment with K6 (2, 4, 8 μM), or L-OHP (8 μM) for 24 h was detected by flow cytometry.

**FigureS4 K6 affects the PI3K-AKT pathway.**


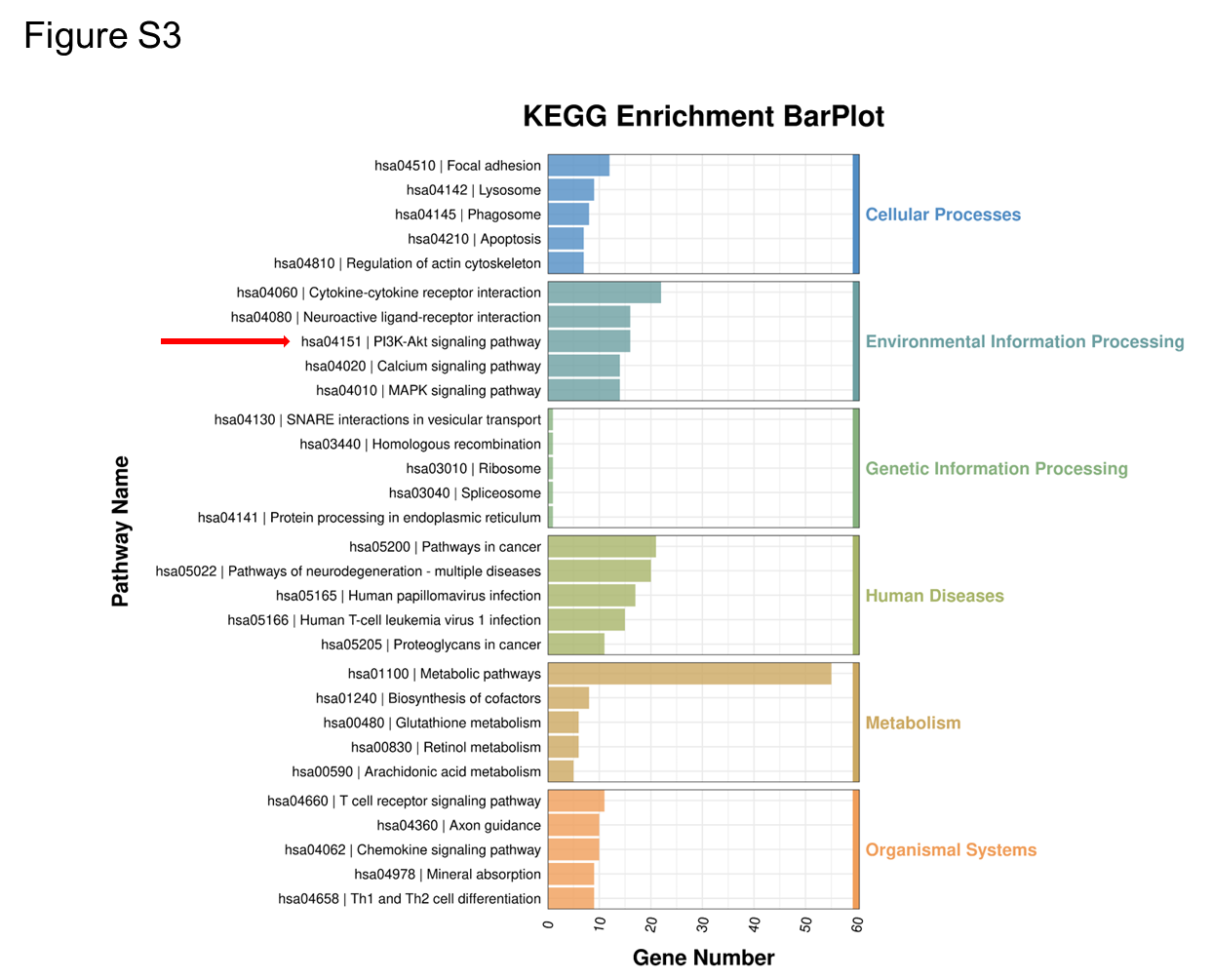


SW480 cells treated with 8 μM K6 for 24 hours and followed by RNA-seq.

**Supplementary Tables**

**Table S1** **Primers sequences for qRT-PCR**

| Target genes | Primer sequences (5'to3') |
| --- | --- |
| c-Myc | F: GTCAAGAGGCGAACACACAA  R: TTGGACGGACAGGATGTATGC |
| WWP1 | F: TGCTTCACCAAGGTCTGATACT  R: GCTGTTCCGAACCAGTTCTTTT |
| GAPDH | F: GGAGCGAGATCCCTCCAAAAT  R: GGCTGTTGTCATACTTCTCATGG |

**Table S2 Routine blood of mice**

| Number of mice | Counts of cells | | | In normal range or not |
| --- | --- | --- | --- | --- |
|  | White blood cells (WBC) | Red blood cells (RBC) | Platelets (PLT) |  |
| Nude-DMSO-1 | 4.83 × 109/L | 10.03 ×1012/L | 836 ×109/L | Yes |
| Nude-DMSO-2 | 4.91 × 109/L | 9.81 × 1012/L | 603 ×109/L | Yes |
| Nude-DMSO-3 | 2.05 × 109/L | 9.38 × 1012/L | 763 × 109/L | Yes |
| Nude-K6-1 | 2.69 × 109/L | 9.39 × 1012/L | 808 × 109/L | Yes |
| Nude-K6-2 | 4.23 × 109/L | 9.80 × 1012/L | 794 × 109/L | Yes |
| Nude-K6-3 | 2.94 × 109/L | 9.38 × 1012/L | 728 × 109/L | Yes |
| C57BL/6-DMSO-1 | 8.77 × 109/L | 8.94 × 1012/L | 1279 × 109/L | Yes |
| C57BL/6-DMSO-2 | 7.35 × 109/L | 9.15 × 1012/L | 1087 × 109/L | Yes |
| C57BL/6-DMSO-3 | 11.57 × 109/L | 9.40 × 1012/L | 1141 × 109/L | Yes |
| C57BL/6-DMSO-4 | 11.22 × 109/L | 9.07 × 1012/L | 987 × 109/L | Yes |
| C57BL/6-K6-1 | 7.37 × 109/L | 8.98 × 1012/L | 1265 × 109/L | Yes |
| C57BL/6-K6-2 | 8.57 × 109/L | 9.03 × 1012/L | 1184 × 109/L | Yes |
| C57BL/6-K6-3 | 6.73 × 109/L | 9.34 × 1012/L | 1200 × 109/L | Yes |
| C57BL/6-K6-4 | 5.52 × 109/L | 8.51 × 1012/L | 837 × 109/L | Yes |
